# Serological diagnosis of soil-transmitted helminth (*Ascaris*, *Trichuris* and hookworm) infections: A scoping review

**DOI:** 10.1371/journal.pntd.0012049

**Published:** 2024-04-04

**Authors:** Sara Roose, Fiona Vande Velde, Johnny Vlaminck, Peter Geldhof, Bruno Levecke

**Affiliations:** Department of Translational Physiology, Infectiology and Public Health, Ghent University, Merelbeke, Belgium; Consejo Nacional de Investigaciones Cientificas y Tecnicas, Fundación Mundo Sano, ARGENTINA

## Abstract

**Background:**

The World Health Organization emphasizes the importance of integrated monitoring and evaluation in neglected tropical disease (NTD) control programs. Serological assays offer a potential solution for integrated diagnosis of NTDs, particularly for those requiring mass drug administration (MDA) as primary control and elimination strategy. This scoping review aims (i) to provide an overview of assays using serum or plasma to detect infections with soil-transmitted helminths (STHs) in both humans and animals, (ii) to examine the methodologies used in this research field and (iii) to discuss advancements in serological diagnosis of STHs to guide prevention and control programs in veterinary and human medicine.

**Methodology:**

We conducted a systematic search in the Ovid MEDLINE, Embase and Cochrane Library databases, supplemented by a Google search using predefined keywords to identify commercially available serological assays. Additionally, we performed a patent search through Espacenet.

**Principal findings:**

We identified 85 relevant literature records spanning over 50 years, with a notable increased interest in serological assay development in recent years. Most of the research efforts concentrated on diagnosing *Ascaris* infections in both humans and pigs, primarily using ELISA and western blot technologies. Almost all records targeted antibodies as analytes, employing proteins and peptides as analyte detection agents. Approximately 60% of sample sets described pertained to human samples. No commercially available tests for *Trichuris* or hookworms were identified, while for *Ascaris*, there are at least seven different ELISAs on the market.

**Conclusions:**

While a substantial number of assays are employed in epidemiological research, the current state of serological diagnosis for guiding STH prevention and control programs is limited. Only two assays designed for pigs are used to inform efficient deworming practices in pig populations. Regarding human diagnosis, none of the existing assays has undergone extensive large-scale validation or integration into routine diagnostics for MDA programs.

## Author summary

To further integrate the monitoring and evaluation of public health control programs against neglected tropical diseases (NTDs), the diagnostic platforms used should ideally be able to screen multiple NTDs in one and the same sample. A stepping stone for more synergies across these programs are blood-based diagnostic assays. Although they have been both developed and implemented to inform public health programs for a number of NTDs, little is known about the progress towards a blood-based diagnostic assays to follow-up on public health programs against intestinal worms. Therefore, we conducted an extensive review of blood-based tests for intestinal worm infections in humans and animals, analysing their progress from assay-development towards routine use in both public and animal health control programs. Our results indicated that, despite many tests are used in research, the use of blood-based assays for guiding control programs against intestinal worms is limited. Only two tests for pigs are currently used to inform deworming programs in pig herds. For human diagnosis, none of the current assays has been extensively evaluated or used for routine diagnosis in control programs.

## Introduction

Today, the world’s most vulnerable communities still bear the heaviest burden of neglected tropical diseases (NTDs), and this has prompted many NTD endemic countries worldwide to take actions to reduce the NTD-attributable disease burden [1]. Depending on the nature of the disease, the established public health interventions involve individual case management or mass drug administration (MDA) to entire communities at risk [2]. While most interventions remain disease specific, important similarities can be identified (e.g., interventions target the same communities [3] and interventions serve multiple NTDs [4]), which provides opportunities to more integrate interventions and to ultimately make better use of resources [2]. Recognizing this, the new 2021–2030 WHO roadmap for NTDs not only makes a plea for more synergies across NTD programs, it also advocates for more integration of NTDs in other well-established public health programs (e.g., HIV/AIDS and malaria) [2].

When aiming for more integrated NTD programs, the diagnostic platforms used should ideally be able to screen multiple NTDs in one and the same sample. Currently, diverse sample types (e.g., stool, urine, blood, skin scrapings, and skin biopsies) are used across various NTDs, employing diagnostic methods primarily reliant on microscopic examination [5]. These methods have several drawbacks, including insufficient performance in low prevalence settings, susceptibility to human error and a limited throughput [5–7]. As a response to this lack of diagnostics, the WHO has published 17 target product profiles (TPPs), which describe the minimal and ideal requirements for various diagnostic needs (e.g., simplicity, performance and price of the test) related to NTD specific use-cases [8]. For individually managed NTDs, the TPPs focus on case confirmation and detection of drug resistance, while those amendable for control through MDA focus on program decisions around starting, scaling down and stopping MDA. When further analysing the TPPs for MDA targeted NTDs (lymphatic filariasis [9], onchocerciasis [10], scabies [11], schistosomiasis [12], soil-transmitted helminthiases [13] and trachoma [14]), it becomes clear that whole blood collected by finger prick is a common sample type, and hence serological diagnostics that use blood fluids (serum or plasma) as specimen are an obvious way forward for more integrated monitoring and evaluation (M&E) of NTDs programs. Moreover, as this is also a common sample type for other infectious diseases (e.g., HIV/AIDS), it provides a unique opportunity to integrate NTDs into existing public health surveillance platforms [15,16]. To date, serological diagnostics are implemented only in MDA programs for lymphatic filariasis and onchocerciasis [2,17,18]. For both NTDs, the WHO defined prevalence thresholds that trigger program decisions to stop MDA [2,17,18]. However, for the remaining four NTDs targeted by MDA efforts, the diagnostic standards remain clinical examination or microscopy, thus hindering inclusion in integrated NTD diagnostics [11–14].

Therefore, this scoping review aims to investigate the landscape of serological assays for infections with soil-transmitted helminths (STHs; *Ascaris*, *Trichuris* and hookworms) focussing on identifying current gaps and key factors needed to advance their application in STH prevention and control programs. The study is guided by three research objectives. Firstly, to list the currently available assays that make use of blood fluids (serum and plasma) as primary specimens to diagnose STH infections. Given the wide prevalence of STH infections in various animal species (e.g., hookworms in dogs [19], *Ascaris* in pigs [20]) our research also seeks to include veterinary tests, recognizing their potential relevance and application in the field of human medicine in the future. Secondly, to examine the methodology used in this research field (e.g., sample sets used and assessments of test performance), and thirdly, to report and discuss advancements in serological diagnosis for soil-transmitted helminthiases to guide prevention and control programs in both veterinary and human medicine.

## Methods

### Search strategy

We opted the broader approach of a scoping review compared to a systematic review, as our aim was to determine the coverage of existing literature on our topic, to examine how research in this field was conducted and to identify knowledge gaps [21]. We did not aim to conduct any formal assessment of the quality of the studies or to critically synthesize and discuss their results. We followed the methodology for scoping reviews outlined in the JBI Reviewer’s Manual [22], and adhered to the reporting guidelines provided in the Preferred Reporting Items for Systematic Reviews and Meta-Analyses extension for Scoping Reviews (PRISMA-ScR) checklist [23] (**S1 Info**). No formal review protocol was registered.

Our search strategy comprised two distinct components: (i) a literature search for published records and (ii) an exploration of commercially available assays and diagnostic services, and patents that either resulted in or could potentially result in commercial assays. All final search strategies are reported in **S2 Info**. The literature search included a systematic search in the Ovid MEDLINE and Embase databases and in the Cochrane Library (24 and 25 September 2022) using database-specific strings based on relevant terms related to serology as well as the combination of commonly used diagnostic assays and the sample type (**S2 Info**). The commercially available serological assays were explored through a Google search using pre-defined keywords (22 September 2022) (**S2 Info**). The patent search was done via Espacenet, a free online service for searching patents and patent applications worldwide (https://worldwide.espacenet.com) (12 October 2022) (**S2 Info**).

### Screening procedure and eligibility criteria

All literature retrieved by the systematic literature search was downloaded to Endnote X9 [24] by one review author (SR), and duplicates were removed. One reviewer (SR) screened the merged search results for potentially relevant records based on title and abstract. Thereafter, two reviewers (SR, PG) reviewed the full-text records for eligibility based on criteria defined during the screening process, independently. The final eligibility criteria are presented in **Table 1**. The two review authors (SR, PG) categorized eligible records into two groups based on their main research focus: group 1 included key papers focusing on serological assay development, while group 2 comprised further relevant research (e.g., studies applying serological assays in epidemiological surveys). Backward and forward reference searching were conducted for all papers in group 1. Throughout the review process, a third author (BL) was responsible for facilitating discussions and reaching consensus in case of disagreement between the reviewers. During these discussions, it became apparent that we had initially overlooked a group of studies related to the use of peptides as analyte detection agents. Consequently, we conducted a supplementary systematic search in the Ovid MEDLINE database (11 October 2022) as reported in **S2 Info**. The additional records identified through backward and forward reference searching of the key papers, as well as the supplementary systematic search for peptide assays, went through the same selection process as the studies that were initially identified through the main systematic search.

**Table 1:** The eligibility criteria used in our scoping review.

Inclusion criteria	Exclusion criteria
Full-text papers and theses, peer reviewed articles	Literature reviews, conference abstracts, editorial letters and comments
Records written in English language	Records written in languages other than English
Records focusing on serological assay development	Records focusing on parasite-specific immune response, host defense against parasites and host-parasite interactions
Epidemiological surveys using serological assays	Case reports using serological assays
	Records on antigen discovery in the scope of vaccine development
	Records assessing cross-reactivity of other serological assays against STHs

The results of the Google and Espacenet search for commercially available tests and patents were summarized using Microsoft Excel (version 16.78). To assess the suitability of each identified commercial test for inclusion in our study, we visited the website of the corresponding manufacturer/distributor to review product specifications, assay protocols, and other relevant information. Inclusion or exclusion was guided by the basic question whether it consisted of a diagnostic assay for detecting STH infection in its natural host, available either as commercial assay or as laboratory diagnostic service. We assessed the eligibility of the patents using the data accessible on Espacenet. This involved a straightforward screening process retaining all patents related to the diagnosis of STHs using serum or plasma as the sample specimen. Screening of commercial assays and patents was performed by one reviewer (SR).

### Data charting

To facilitate data charting of the literature search results, a data charting form was designed upon agreement by three review authors (SR, PG, BL). This form was developed through both iterative discussions and pilot testing on several key records. Data charting was completed by one review author (SR), but discussed at length with two other review authors (PG, BL). The final data charting form included: (i) the year of publication, (ii) details about authors and research group, (iii) the primary aim of the study (e.g., assessment of seroprevalence), (iv) the target species (e.g., *Ascaris*) and target disease in the case of *Ascaris* (intestinal parasitosis or larva migrans syndrome (LMS) [25,26]), (v) the test species (e.g., human), (vi) the study population, (vii) the analyte detection agent used (e.g., somatic antigens of adult *Ascaris*), (viii) the test technology principle applied (e.g., enzyme-linked immunosorbent assay (ELISA)), (ix) the target analyte detected (e.g., anti-*Ascaris* IgG4), (x) source of the assay (in-house or commercially available), and (xi) specific test parameters (e.g., cut-off value, sensitivity and specificity). To ensure clarity and transparency, we have provided descriptions of the key theoretical terms used in this scoping review in **Table 2**.

**Table 2:** Glossary of the key terms used in this scoping review.

Term	Description
**Serological diagnosis**	Diagnosis that involves the use of assays that specifically analyse blood fluids (serum and plasma) as the primary sample type.
**Target species**	The soil-transmitted helminth species (*Ascaris*, *Trichuris* or hookworms) for which the assay is primarily designed.
**Test technology principle**	The laboratory methodology upon which the specific diagnostic assay is built (e.g., ELISA).
**Target analyte**	The biomolecule that the given diagnostic assay is designed to identify, measure or quantify within a sample (for example parasite-specific antibodies).
**Analyte detection agent**	The component of the assay used to identify, capture or quantify the presence/absence of a particular target analyte within a sample. It interacts specifically with the target analyte (for example parasite antigen).
**Test species**	The particular species from which blood serum or plasma samples were subjected to analysis.

We used the same data charting form to screen all commercially available tests, except for the year of publication, the primary aim of the study, the study population and the source of the assay. Meanwhile, we included information on the name of the test and its manufacturer. Companies were contacted through e-mail to obtain all necessary data. At last, we aimed to extract the same data from the retrieved patent documents. Data management and tabulation were performed using Microsoft Excel (version 16.78). Data visualization of the charting results was done using R Studio (Version 2023.03.0+386) [27] and Microsoft PowerPoint (version 16.78).

### Synthesis approach

We synthesised all data and directed our analysis towards six key aspects:

**i. Target species and temporal trends of the published literature**: we investigated target species and the temporal trends of the published literature.
**ii. Characteristics of the assays used for serodiagnosis of STHs**: we delved into the characteristics of reported assays. For this, we summarised the technology principles employed, the specific target analytes, and the agents used for analyte detection.
**iii. Characteristics of described sample sets**: we summarized the sample sets described in the records, documenting the test species, origin of samples and size.
**iv. Evaluation of serodiagnostic assay performance**: we examined how diagnostic cut-off values, and the parameters diagnostic sensitivity and specificity were defined by the researchers to evaluate the diagnostic performance of the assay.
**v. Advancements in serological diagnosis of soil-transmitted helminthiases**: we reflected on progress in serological diagnosis of STHs. For this research question, we defined the most advanced stage as the point where the assay is routinely used to guide STH prevention and control programs in veterinary or human medicine. This stage represents the intended use case as defined in the WHO’s target product profiles for human STHs [13]. We categorized all included assays into four distinct stages, including assay development (Stage 1), cut-off establishment (Stage 2), research use (Stage 3), and routine implementation to guide STH prevention and control programs (Stage 4). The criteria applied to determine the advancement stage of the assays are summarized in **Table 3**.
**vi. Patents and commercially available tests**: we summarized the outcomes of searches for commercially available assays and patents, and we evaluated the stage(s) where these commercial assays went through, applying the criteria described in **Table 3**.

**Table 3:** The criteria applied to determine the four assay advancement stages.

Advancement stage	Criteria
**Stage 1: assay development**	▪ The discovery and investigation (e.g., epitopes, protein structures, and glycans) of new detection agents ▪ The assessment of the detection agent’s performance using a particular technology principle ▪ The determination of the optimal target analyte (e.g., isotype) and its dynamics over time ▪ The evaluation of cross-reactivity
**Stage 2: cut-off establishment**	▪ The establishment of a diagnostic cut-off to distinguish between positive and negative samples
**Stage 3: research use**	▪ The use of assays in endemic settings to investigate a range of specific epidemiological questions, such as determining the prevalence in a population, examining how this prevalence varies across different demographic factors (e.g., age and sex), identifying risk factors for infection, investigating the infection dynamics and transmission patterns, assessing the effectiveness of different control strategies, identifying how infections interact with other health outcomes (e.g., malnutrition and other infections) ▪ Investigation of the potential of assays to be used in epidemiological studies
**Stage 4: routine implementation to guide STH prevention and control programs**	▪ The use by the intended user in target populations to guide prevention and control programs (e.g., used by veterinarians in pig herds to assess the impact of deworming, used by program managers to guide MDA programs)

## Results and discussion

### Search strategy

**Fig 1:**
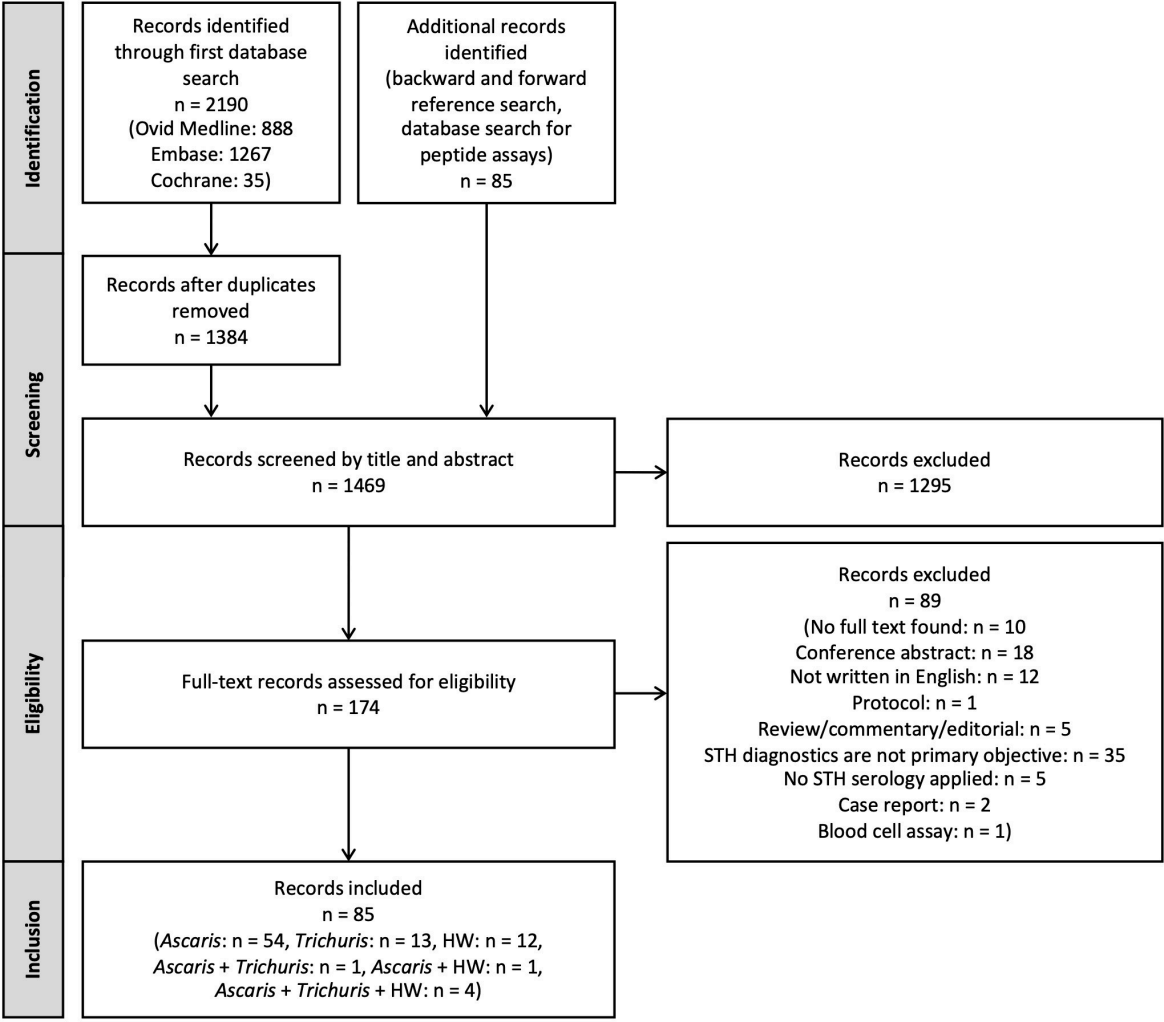
Flow chart of the literature search and screening procedure. STH: soil-transmitted helminth, HW: hookworm.

Our search strategy comprised two distinct components: (i) a literature search for published records and (ii) an exploration of commercially available assays and diagnostic services, and patents that either resulted in or could potentially result in commercial assays. **Fig 1** presents the literature search and screening procedure that was employed for the published records only. This literature search involved two systematic database searches as well as a reference search, which together yielded a total of 1,469 unique records. After screening these records by title and abstract, 174 records were found eligible. Finally, a total of 85 records were included in this scoping review of which a detailed summary is provided in **S3 Info**, including (i) the year of publication, (ii) details on the title, journal and authors, (iii) the advancement stage applying the aforementioned criteria (**Table 3**), (iv) the test species (e.g., humans), (v) details on the study population, (vi) the analyte detection agent used (e.g., somatic antigens of adult *Ascaris*), (vii) the assay technology principle applied (e.g., ELISA), (viii) the target analyte detected (e.g., IgG4), (ix) the source of the assay (in-house or commercially available), and (x) specific assay parameters (cut-off value, diagnostic sensitivity and specificity). Given the distinct biological differences in STH species, we have clustered these details on an STH species level. For assays targeting *Ascaris*, the list also includes (xi) the target disease, either intestinal parasitosis or LMS [25,26].

The second component of our search strategy yielded the following outcomes. We were unable to identify any commercially available tests for *Trichuris* or hookworm infections. However, in the context of *Ascaris*, we identified a minimum of seven different tests for natural host species currently available on the market. Conducting the patent search resulted in 42 records, which were manually assessed to determine eligibility. Among these, three patents were found to be relevant to our research questions.

### Target species and temporal trends of the published literature

**Fig 2:**
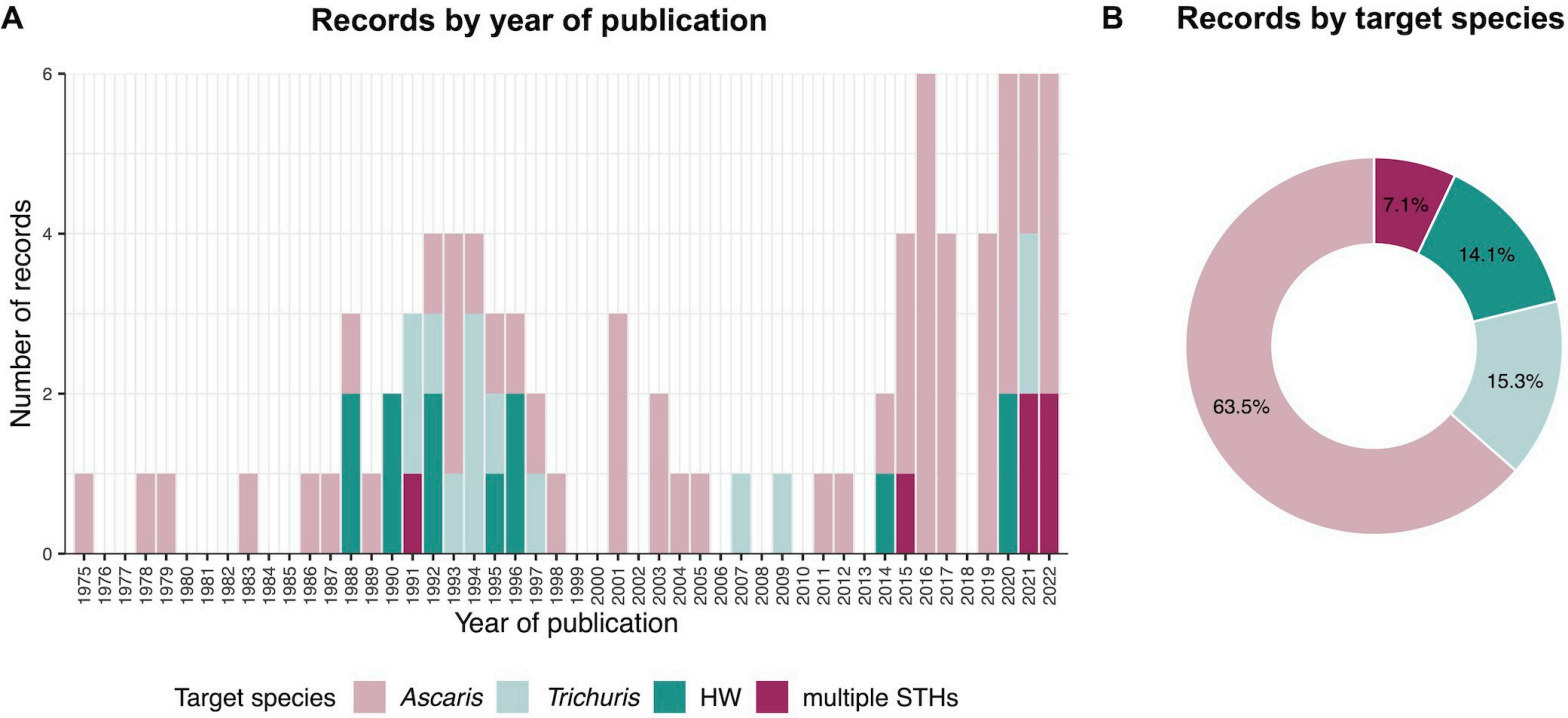
Target species and year of publication of the 85 records included in our review. HW: hookworm, STH: soil-transmitted helminth. **S3 Info** provides an overview of the 85 records used to create the figures.

**Fig 2** illustrates two key aspects of the 85 included records: the year of publication (**Fig 2A**) and the target species (**Fig 2B**). A significant portion (79 records; 92.9%) concentrated on a single STH species. Among these, 54 records focused on *Ascaris*, 13 records focused on *Trichuris*, and 12 records focused on hookworms. In contrast, only six records (7.1%) targeted multiple STH species. Among these, four records examined all three STHs, one record focused on *Ascaris* and *Trichuris*, and one record focused on *Ascaris* and hookworms.

Our analysis revealed a distinct pattern in the publication timeline. Before 1987, retrieved records only focused on *Ascaris*. Between 1988 and 1998, we observed an initial surge of records on all three STH species. Notably, the majority of records in this period focusing on *Trichuris* were attributed to a single research group, unlike records involving *Ascaris* and hookworms (**S3 Info**). From 1998 to 2014, there was a relatively limited and skewed research output. However, since 2014, we have observed a renewed interest in the development of serological assays, with an emphasis on diagnosing multiple STH infections simultaneously.

### Characteristics of the assays used for serodiagnosis of STHs

**Fig 3:**
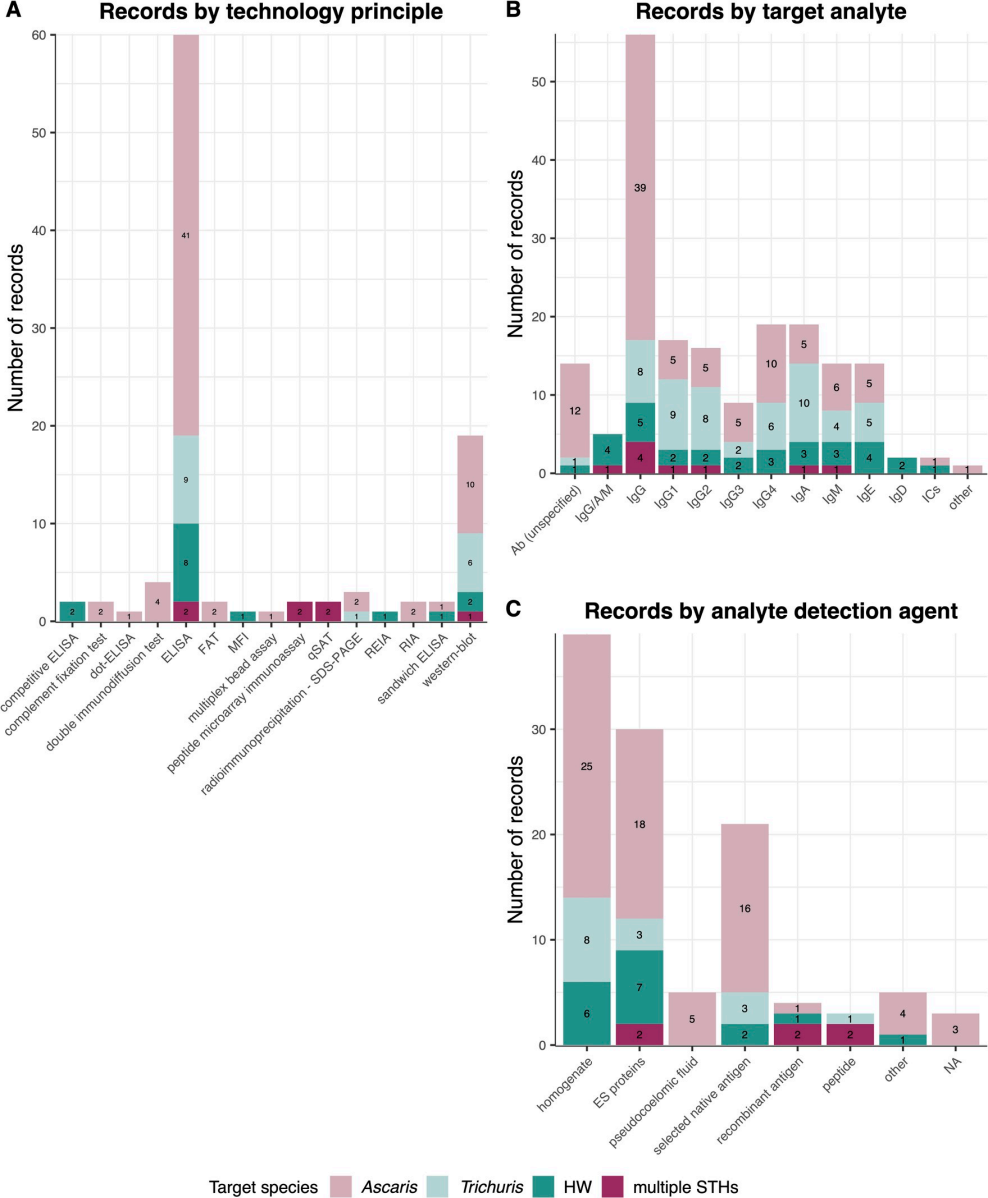
Characteristics of the assays used for serodiagnosis of STHs. The number of records by technology principle applied (**panel A**), target analyte detected (**panel B**) and analyte detection agent used (**panel C**). The target analyte refers to the specific analyte that a test is designed to detect or measure, while the analyte detection agent refers to the product used in the test to investigate the presence of the target analyte. The color indicates the STH species targeted in each record. In cases where a record applied multiple options, the records was included in the count of all relevant options. FAT: fluorescent antibody test, ELISA: enzyme-linked immunosorbent assay, MFI: multiplex flow immunoassay, qSAT: quantitative suspension array technology, SDS-PAGE: sodium dodecyl sulfate polyacrylamide gel electrophoresis, REIA: reverse enzyme immunoassay, RIA: radioimmunoassay, Ab: antibody, Ig: immunoglobulin, IC: immunecomplex, ES: excretory/secretory, HW: hookworm, STH: soil-transmitted helminth. **S3 Info** provides an overview of the 85 records used to create the figures.

**Fig 3** summarises the 85 records by the applied technology principle (**Fig 3A**), the target analyte detected (**Fig 3B**) and the analyte detection agent used (**Fig 3C**). Our analysis also considered the landscape of serodiagnostic research over time, graphically presented in **S4 Info**, to gain insights into how these three assay characteristics have evolved throughout the years.

#### Technology principle

**Fig 3A** highlights that ELISA (used in 60 records) and western blot (used in 19 records) are the most applied technologies in research on serodiagnosis for STHs, employed for over five decades as shown in **S4 Info**, from the 1970s to 2022. Note that, when referring to ELISA in this context, it specifically pertains to the indirect ELISA type. Additionally, variants of the indirect ELISA principle, such as reverse enzyme immunoassay (REIA), sandwich ELISA, and multiple-dot ELISA, have also been explored [28–30]. Direct and indirect fluorescent antibody tests (FAT) [31,32], radioimmunoassays (RIA) [33,34], complement fixation tests [35,36], and double immunodiffusion tests [30,34,36,37], were employed until 2003, after which ELISA and western blot became the predominant techniques together with new technologies that appeared on the scene (**S4 Info**). Among these new technologies, the multiplex bead assay [38], peptide microarray immunoassays [39,40] and quantitative suspension array technology (qSAT) assays [41,42] represent examples of multiplex assays. These advanced technologies have been applied in studies focused on serodiagnosis for multiple STH species (**S3 Info**). Notably, an interesting distinction is seen in **Fig 3A**, showing technological diversity within different STH targets. Records on *Trichuris* and hookworms have a more limited range of technology principles. Conversely, research on *Ascaris* demonstrates a broader spectrum of technologies used.

#### Target analyte

As shown in **Fig 3B**, apart from three, all records report human or animal antibodies as target analytes. **S4 Info** indicates that early research did not always differentiate between antibody isotypes, possibly due to the unavailability of specific conjugates. For the detection of *Ascaris* infections, IgG appears to be the preferred target analyte for pigs, whereas IgG4 has become the most interesting target for humans [43–47] (**S3 Info**). For *Trichuris*, the most recent studies in humans focus on total IgG [40,48] (**S4 Info**). For hookworms, researchers have strongly debated about the most appropriate isotype, with some advocating for IgE [49] or IgG4 [50,51], but recent research has focused mainly on total IgG, IgG4, and IgM [40,52,53] (**S4 Info**). The three remaining records investigated the diagnostic value of detecting immune complexes (ICs) (i.e., antigen-antibody complexes) [29,54], and *Ascaris* proteins [33] in blood fluids.

#### Analyte detection agent

In **Fig 3C**, we have grouped the analyte detection agents in seven categories: homogenate, excretory/secretory (ES) antigens, pseudocoelomic fluid, selected native antigen, recombinant antigen, peptide or other. Detailed descriptions of the analyte detection agents can be found in **S3 Info**. It was observed that the majority of records focused on antibodies as target analytes, and as a result, most analyte detection agents were proteins and peptides that bind to or interact with the target analyte to enable its detection and/or quantification. It appeared that over 50 years of research, most assays relied on protein mixtures, particularly homogenates and ES antigens of larvae or adult worms (**S4 Info**). For *Ascaris*, researchers also explored the potential of its pseudocoelomic fluid, although this was not the case for *Trichuris* or hookworms, likely due to practical considerations related to the size of the worms (*Trichuris*: up to 5 cm; hookworms: up to 2 cm; *Ascaris*: up to 40 cm) [36,37,55–57]. In recent years, there has been a shift towards studies focusing on specific proteins (e.g., cuticular collagen of *Necator americanus* [58], native *Ascaris suum* haemoglobin [47,59,60]) rather than antigen mixtures (**S4 Info**). In addition, recombinantly produced forms of specific proteins [41,42,53] offer advantages such as reproducibility and sustainability as a resource when compared to native antigen preparations. Peptide research [39,40] is particularly interesting as it allows for the identification of specific epitopes and serves as a stepping stone for the development of multiplex diagnostic assays. The ‘other’ category in our analysis includes parasite cross-sections [31] and the use of anti-parasite serum (e.g., rabbit anti-*A*. *suum* serum [33], chicken anti-*A*. *suum* IgY [29,54]), as well as hatching fluid [56].

### Characteristics of described sample sets

**Fig 4:**
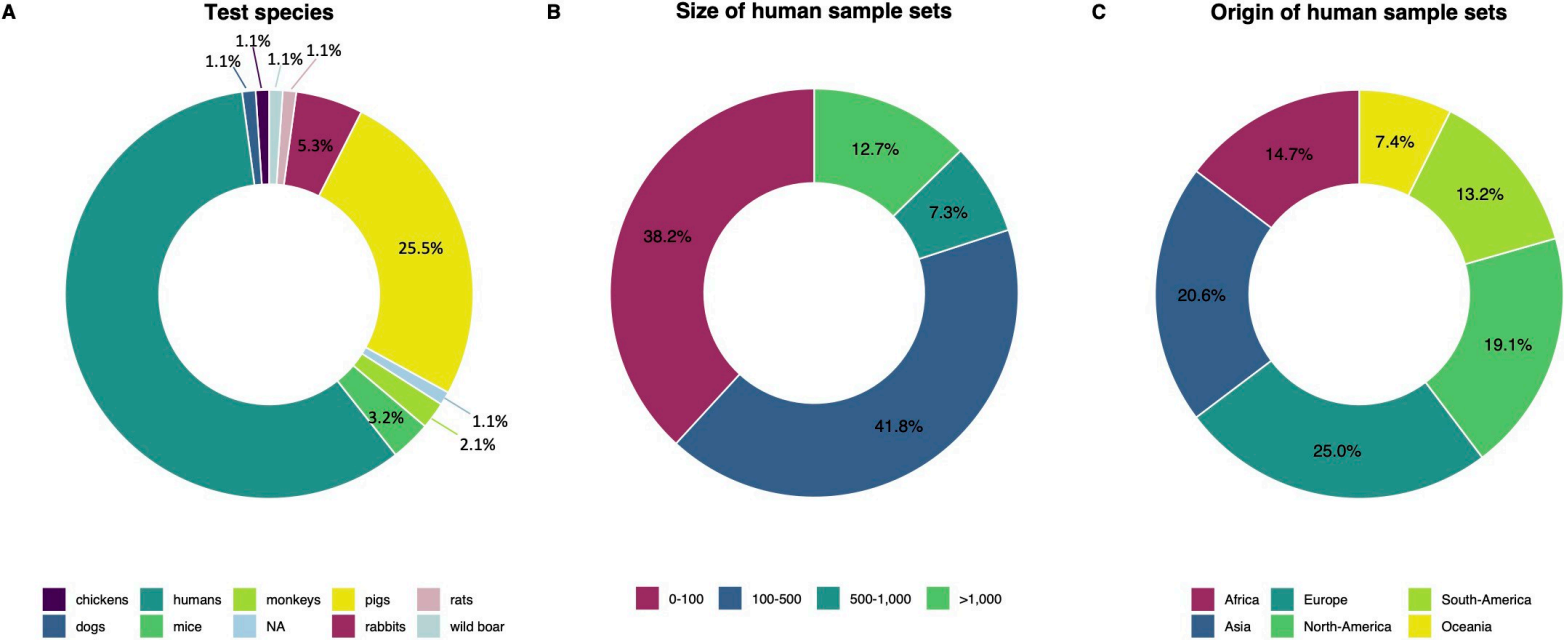
Characteristics of test species and human sample sets. **Panel A** displays the proportion of sample sets that involved a particular test species. **Panel B** indicates the size of the human sample sets. **Panel C** provides insights into the origin of the human sample sets. Certain sample sets were re-used and therefore reported in multiple records, resulting in multiple counts in panels A and B. To create panel C, we considered that a reported sample set might contain samples from different origins (e.g., positive samples from Africa, negative samples from Europe), and treated these as distinct human sample sets in our origin analysis. **S3 Info** provides an overview of the 85 records used in the analyses.

Of all the sample sets described in the 85 included records, 58.5% covered human samples as shown in **Fig 4A**. The rationale behind testing non-human samples mainly lies in the application of serodiagnosis for animals that are natural hosts for STH species, such as pigs in the context of *Ascaris* (25.5% of all sample sets described). Additionally, some studies used experimentally infected animals or laboratory animals for assays in development (e.g., rabbits (5.3%) and mice (3.2%)). One of the records reports a setback in the production of an effective multiplex flow immunoassay for hookworms and consequently, no sample set was reported for further investigation [52].

Regarding human sample sets, **Fig 4B** indicates that sample sizes ranged from six to 6,718 individuals, and **Fig 4C** provides insights into their origin. The majority of endemic samples were collected in Asia and North-America. However, the latter may partly stem from the reuse of the same sample sets, especially in older records, for investigating various research questions [61–68] (**S3 Info**). Remarkably, despite the use of sample sets from Europe as non-endemic negative controls, there were also seven studies that assessed seroprevalence in European regions (e.g., The Netherlands and Austria) [69–75].

### Evaluation of serodiagnostic assay performance

The performance parameters (diagnostic sensitivity and/or specificity) were reported for 26 in-house assays and they are summarized in **Table 4** (full details can be found in **S3 Info**). Overall, the range in reported diagnostic sensitivity and specificity equalled 11.8% - 100% and 0% - 100%, respectively. The observed variation in performance is not unexpected and can already be explained by inherent differences in performance across the specific antigens used [42,53,76], the antibody isotype [51,76], the diagnostic platform [35] and the test species [77]. However, it is important to also acknowledge the methodological differences, such as the sample sets [77], the methodology to determine the cut-off [42,78], and the definition of the reference standard [57]. The sample sets used to define these parameters, show significant variability in size and were collected from both human and animal hosts, as well as immunised animals. Four different methods were employed to define cut-off values, including both direct approaches (the mean plus a multiple of the standard deviation of the values obtained in negative samples or using a multiple of the value observed in negative samples), and more advanced methods (Gaussian mixture models and receiver operating characteristic curves). As expected, the most frequently applied reference standard for STH infection was the presence of worm eggs in stool, which were detected through different microscopic methods (e.g., McMaster, Kato-Katz thick smear, zinc sulphate flotation and formol ether concentration). Alternative reference standards were the presence of liver white spots or adult worms, experimental infections, or qPCR data. Generally, the absence of a true gold standard for STH infections is an important obstacle to accurately assess the diagnostic performance. Detecting eggs is only possible when mature adult worms are present, and although microscopic methods are widely used, they come with inherent limitations in terms of sensitivity and specificity. The assessment of liver white spots, an immunological response of the host during larval migration through liver tissue, is subjective and prolonged exposure of individuals to the parasite can negatively impact the test’s sensitivity. When it comes to diagnosing *Ascaris* LMS, there are even no alternative diagnostic methods available [71]. On the contrary, animals subjected to experimental infection with a single STH species can represent the ultimate gold standard [59,79–83]. However, when interpreting diagnostic sensitivity and specificity of tests validated through experimental infections, it is crucial to consider two key elements of these experiments: (i) the infection dose (e.g., seroconversion occurs more rapidly with higher parasite infection doses) and (ii) the duration of infection (e.g., prolonged exposure can lead to a higher seroconversion rate) [56]. Although using extremely low infection doses administered as trickle infections to mimic natural infections is a potential approach, the representativeness of these infections still remains uncertain, as there are currently no data available on actual infection doses in natural cases, such as the quantity of eggs or larvae children are exposed to when living in endemic regions. Another important aspect when evaluating assay performance is the potential for biases in study populations. When screening individuals or animals for STH infections, it is important to acknowledge that these subjects might have been exposed to other (non-)parasitic infections, adding complexity into the diagnostic process. A strategy to manage this complexity is to identify and document other infections and include the samples in the negative control group, allowing the evaluation of cross-reactivity [51,78]. Nevertheless, this approach assumes that all other infections are easily distinguishable from the targeted STH infections. In reality, this can be challenging, especially if they trigger similar immune responses. Additionally, it assumes that all relevant co-infections are known and documented, which is rarely the case.

**Table 4:** Summary of all the in-house assays for which diagnostic sensitivity and specificity were assessed. Records are clustered based on target species, technology principle and analyte detection agent. HW: hookworm, LMS: larva migrans syndrome, FAT: fluorescent antibody test, ELISA: enzyme-linked immunosorbent assay, REIA: reverse enzyme immunoassay, qSAT: quantitative suspension array technology, Ig: immunoglobulin, ES: excretory/secretory, SD: standard deviation, STH: soil-transmitted helminth, ROC: Receiver Operating Characteristic, Ref: reference.

Target species	Technology principle	Analyte detection agent	Target analyte	Samples tested	Number of samples (range)	Reference standard for STH infection	Cut-off value	Diagnostic sensitivity	Diagnostic specificity	Ref
***Ascaris***	FAT	Homogenate	Antibodies	Immunized animals (rabbits), natural host animals (dogs, rats), humans	100–200	NA	NA	100%	91–95%	[31]
***Ascaris***	FAT	Homogenate	Antibodies	Humans	400–500	(Copro)microscopy	Mean + X SD	95–98%	95–98%	[32]
***Ascaris***	FAT	Other	Antibodies	Immunized animals (rabbits), natural host animals (dogs, rats), humans	100–200	NA	NA	100%	91–95%	[31]
***Ascaris***	ELISA	Homogenate	Antibodies	Pigs	200–300	Liver white spots	NA	“about 50%”	“about 50%”	[35]
***Ascaris***	ELISA	Homogenate	IgG	Humans	300–400	(Copro)microscopy	Mean + X SD	76–87%	NA	[84]
***Ascaris***	ELISA	Homogenate	IgG	Immunized animals (pigs), natural host animals (pigs)	100–200	(Copro)microscopy and/or adult worms	NA	89.74–100%	0–2.43%	[57]
***Ascaris***	ELISA	Homogenate	IgG	Natural host animals (pigs)	800–900	Experimental infection	ROC analysis	≧ 90%	99%	[79]
***Ascaris***	ELISA	ES proteins	Antibodies	Natural host animals (pigs)	100–200	Liver white spots	Mean + X SD	97%	89%	[55]
***Ascaris***	ELISA	ES proteins	IgG, IgG1, IgG2, IgG3, IgG4, IgA, IgM, IgE	Humans	100–200	(Copro)microscopy	Mean + X SD	11.8–100%	100%	[76]
***Ascaris* (LMS)**	ELISA	ES proteins	IgG	Humans	100–200	NA	Mean + X SD	NA	76%	[70]
***Ascaris***	ELISA	Pseudocoelomic fluid	IgG	Immunized animals (pigs), natural host animals (pigs)	100–200	(Copro)microscopy and/or adult worms	NA	91.1–100%	0–2.43%	[57]
***Ascaris***	ELISA	Pseudocoelomic fluid	Antibodies	Natural host animals (pigs)	100–200	Liver white spots	Mean + X SD	84%	94%	[55]
***Ascaris***	ELISA	Selected native antigen	IgG	Natural host animals (pigs)	± 1,000	Experimental infection	ROC analysis	99.5%	100%	[59]
***Ascaris***	ELISA	Selected native antigen	IgG	Immunized animals (pigs), humans	50–100	Pigs: experimental infection Humans: (copro)microscopy	ROC analysis	92.0–98.4%	90.0–95.5%	[77]
***Ascaris***	Sandwich ELISA	Other	Immune complexes (IgG)	Humans	50–100	(Copro)microscopy	ROC analysis	80%	90%	[29]
***Ascaris***	Complement fixation test	Homogenate	Antibodies	Pigs	200–300	Liver white spots	NA	50–73%	77%	[35]
***Ascaris* (LMS)**	Western blot	ES proteins	IgG	Humans	100–200	NA	NA	100%	95%	[71]
***Trichuris***	ELISA	Selected native antigen	Antibodies	Natural host animals (pigs, dogs)	0–50	Experimental infection	3 x value	"high"	100%	[85]
**HW**	ELISA	Homogenate	Globulin	Humans	50–100	(Copro)microscopy	Mean + X SD	43–93%	NA	[78]
**HW**	ELISA	Homogenate	IgG	Humans	50–100	(Copro)microscopy	Mean + X SD	66%	NA	[53]
**HW**	ELISA	ES proteins	Globulin	Humans	50–100	(Copro)microscopy	Mean + X SD	93–97%	NA	[78]
**HW**	ELISA	Recombinant antigen	IgG	Humans	50–100	(Copro)microscopy	Mean + X SD	41–82%	NA	[53]
**HW**	REIA	ES proteins	IgE	Humans	50–100	(Copro)microscopy	Mean + X SD	100%	96%	[28]
**HW**	Sandwich ELISA	Other	Immune complexes (IgG)	Humans	50–100	(Copro)microscopy	ROC analysis	90%	86.7%	[54]
**HW**	Western blot	Selected native antigen	IgG, IgG1, IgG2, IgG3, IgG4	Humans	50–100	(Copro)microscopy or adult worms	NA	75%	84–100%	[51]
**STHs**	qSAT	Recombinant antigen	IgG	Humans	700–800	(Copro)microscopy and qPCR	Gaussian mixture models, mean + X SD, ROC analyses	See record for all combinations	See record for all combinations	[42]

### Advancements in serological diagnosis of soil-transmitted helminthiases

**Fig 5:**
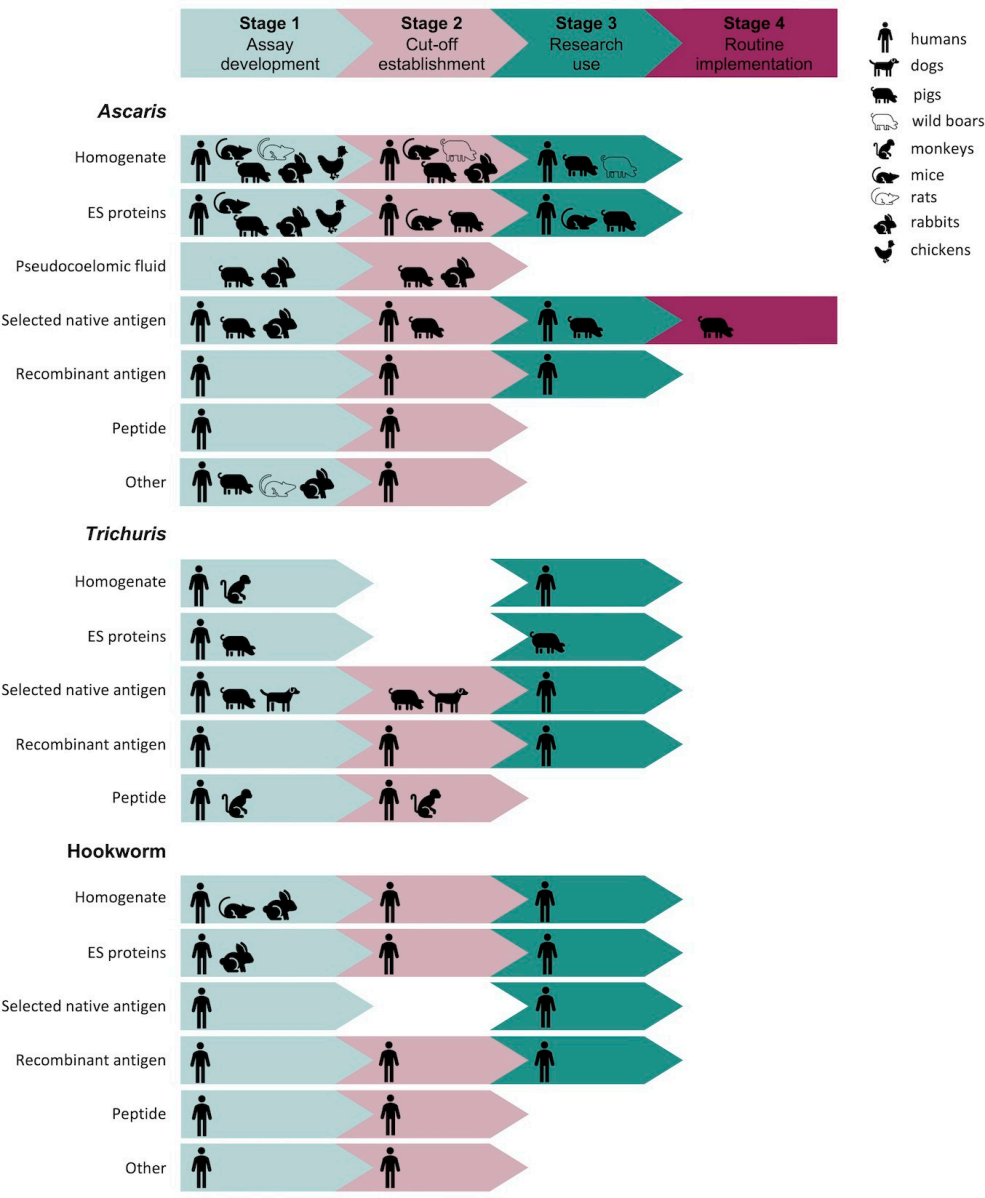
Summary of advancements in the serological diagnosis of soil-transmitted helminthiases. Assays/records are listed based on the described analyte detection agent. The icons represent the species comprising the reported sample sets. A comprehensive description of the four stages can be found in **Table 3** and **S5 Info** provides a systematic overview of the records used to create this figure.

**Fig 5** offers a summary of advancements in the serological diagnosis of soil-transmitted helminthiases. **S5 Info** provides an overview of the records used to create **Fig 5**. All records were listed based on the described analyte detection agent because of the essential role played by the discovery of these agents in the development of novel tests. Typically, a specific analyte detection agent is tested in several test technology principles, and assay-optimization might include assessment of different target analytes. For instance, researchers may initially examine their discovered analyte detection agent (e.g., a recombinant antigen) in western blotting, thereafter transition to ELISA, and subsequently refine their test by the detection of specific target analytes (e.g., immunoglobulin isotypes). Three records were excluded from **Fig 5** ([69], [86] and [87]) because they made use of commercially available tests for which the analyte detection agent was unspecified.

Based on our analysis, four interesting observations were made. First, samples from both laboratory animals (e.g., mice) and natural hosts (e.g., dogs) were used for the development of the assay (Stage 1) and the establishment of diagnostic cut-offs (Stage 2), while research use (Stage 3) was mostly observed in porcine and human populations. Additionally, a broader range of test species was employed for *Ascaris*-related research. A second remarkable finding is the fact that certain records used assays in endemic settings without establishing diagnostic cut-offs. A third significant observation is the correlation between a higher percentage of records focusing on *Ascaris* and a greater number of assays progressing to Stage 3, as compared to those related to *Trichuris* and hookworm infections. Further investigation of **S3 Info** and **S5 Info** revealed that four specific *Ascaris* assays show recurrent use across several recent epidemiological records (Stage 3). The first of these notable assays is an ELISA coated with ES proteins from migrating larvae, originally developed based on an assay for the diagnosis of LMS due to *Toxocara* [88]. This ELISA was described by Pinelli and colleagues as the most recommended serological assay for the diagnosis of *Ascaris* LMS [70]. A second notable assay for diagnosing LMS, is an immunoblot developed by Schneider et al. (2015), also relying on larval ES material [71]. A third assay, first described by Vlaminck et al. (2012) for intestinal ascariasis in pigs, is an ELISA based on native *A*. *suum* haemoglobin [59]. Finally, an ELISA using homogenate from *A*. *suum* lung stage larvae, introduced by Vandekerckhove et al. (2017), completes the set of assays repeatedly used in the included records [79]. A last and important observation made from **Fig 5** is the fact that none of the literature records included in our analysis mentioned the routine implementation of assays to guide STH prevention and control programs. Nonetheless, there is evidence suggesting that certain commercial assays have progressed to this stage, as we discuss in more detail below.

### Patents and commercially available tests

Through the patent search, we identified 42 records that were subsequently assessed for eligibility. Three patents were related to our research questions (**S6 Info**). However, to the best of our knowledge, none of the patents have resulted in successful novel diagnostic tests with present-day applications.

We were unable to identify any commercially available tests for *Trichuris* or hookworms. In the case of *Ascaris*, there are at least seven different tests commercially on the market, all are ELISA assays (**Table 5**). Two of these tests are intended for the use in pigs. The SERASCA assay, based on native *Ascaris* haemoglobin, was developed at Ghent University (Belgium) and is currently marketed as laboratory diagnostic service for the diagnosis of *Ascaris* infection pressure in fattening pigs. Based on the SERASCA, the ELISA kit Monoscreen AbELISA *Ascaris suum* (Bio-X Diagnostics) is commercially available. Published research papers provide a comprehensive description of how the SERASCA assay was developed and validated [59,60]. The performance of the Monoscreen ELISA was calculated using the SERASCA assay as reference. Preceding sections of this scoping review incorporated five records using this assay as an in-house research tool [59,60,79,89,90]. In addition, five records made use of the commercial versions of this ELISA to evaluate seroprevalence of *A*. *suum* infection in Austria [91], France [92], Greece [93], Finland [94] and China [20] in the framework of epidemiological research (Stage 3 in **Fig 5**). Online information for both tests explicitly state their diagnostic purpose, which is to estimate the *Ascaris* infection intensity in a pig herd. This underscores that both tests have progressed to the advanced phase of routine application to guide prevention and control programs by the intended users, namely veterinarians. To the best of our knowledge, these two assays are the only ones that have progressed to Stage 4.

**Table 5:** Overview of the commercially available ELISAs for soil-transmitted helminthiases. Records were retrieved by a Google keyword search. A limitation of our search strategy is that records that were buried deeper in the search rankings are potentially omitted. However, we deem our approach comprehensive and inclusive for our study’s specific objectives. NA: information not available. ELISA: enzyme-linked immunosorbent assay.

Test species	Test name	Manufacturer	Commercial kit/lab diagnosis	Analyte-detection agent	Target analyte	Diagnostic sensitivity	Diagnostic specificity
Pigs	SERASCA-Test	Laboratory of Parasitology—Ghent University	Lab diagnosis	Native *Ascaris* suum hemoglobin protein	IgG	99.5%	100%
	MonoScreen AbELISA *Ascaris suum*	Bio-X Diagnostics S.A.	Commercial kit	Native *Ascaris suum* hemoglobin protein	IgG	Relative to SERASCA: 81,2%	Relative to SERASCA: 86,1%
Humans	NovaLisa *Ascaris lumbricoides* IgG ELISA (& Human Anti-*Ascaris lumbricoides* IgG ELISA Kit)	NovaTec Immundiagnostica (& Abcam plc.)	Commercial kit	*A*. *lumbricoides* crude extract	IgG	>95%	>95%
	Human *Ascaris lumbricoides* IgG antibody ELISA kit	Arigo Biolaboratories Corporation	Commercial kit	*Ascaris* crude extract	IgG	100% (95% CI: 47.82% - 100%)	95.0% (95% CI: 87.69% - 98.62%)
	*Ascaris* IgG ELISA	Bordier Affinity Products SA	Commercial kit	*Ascaris* coelomic soluble antigens	IgG	1 study: 81%	4 studies: from 57% to 98%
	Human anti-*Ascaris lumbricoides* antibody (IgM) ELISA Kit	MyBioSource, Inc.	Commercial kit	NA	IgM	NA	NA
	Human anti-ascaris lumbricoides antibody(IgM) Elisa Kit	AFG Bioscience	Commercial kit	Recombinant protein: ACJ03764	IgM	≥82.1%	≥85.7%

Five ELISA kits retrieved by our search are developed for the detection of ascariasis in humans. The NovaTec ELISA is distributed by at least six distributors, indicating a relatively broad availability of this assay in the market of Europe and North America. The detection agents used in the assays are homogenate (crude extract), pseudocoelomic fluid, or a recombinant protein, although this information was not provided by all companies. The two ELISA kits based on *A*. *lumbricoides* crude extract (NovaTec Immundiagnostica and Arigo Biolaboratories Corporation) are equipped with a cut-off control sample and the datasheets give clear instructions on the interpretation of results. These sheets report a diagnostic sensitivity and specificity of more than 95%, though it is not mentioned how this was determined. On the contrary, the website of the third human kit, Bordier Affinity Products SA, does provide data on the sample sets used to define sensitivity and specificity, however the size of the sample sets used is relatively small (minimum: 27 samples, maximum: 181 samples) and information on the reference standard for infection and the origin of the samples is limited. Detailed information on the performance of the fourth kit (MyBioSource, Inc.), detecting anti-*Ascaris* IgM, is absent. The fifth human kit (AFG Bioscience), also detecting IgM, has a reported diagnostic sensitivity and specificity of ≥82.1% and ≥85.7% respectively. The unique feature of this kit is the fact that the ELISA plate is coated with a recombinant protein of *Ascaris*, of which the sequence is referring to *Ascaris* Ag1 (NCBI accession number: ACJ03764), one of the twenty most abundant proteins in *Ascaris* ES products [95]. Our literature search strategy retrieved four records in which commercially available assays were employed [69,74,86,87]. In one record, Lassen and colleagues used the NovaLisa *Ascaris lumbricoides* IgG ELISA for epidemiological research (Stage 3 in **Fig 5**) [74]. Unfortunately, in the other three cases, though the distributors were mentioned, it was unclear which specific assays were used. This can be attributed to changes in names or reference numbers, current unavailability of the kits, or changes in manufacturers.

In contrast to the veterinary kits, the origin of all five human assays remains ambiguous, as it is unclear whether they are the result of academic research endeavours or if they were developed within the commercial sector. We decided not to include information on the intended use claim of the tests (research use only or medical purposes) to avoid potential confusion related to different definitions and guidance documents (for example between Europe and the USA). However, all available documents imply that these assays are intended for individual patient diagnostics rather than for assessing prevalence within larger populations. The descriptions of the antigens used to coat the ELISA plates are poorly defined, and detailed methodologies regarding their production are absent. While diagnostic sensitivity and specificity are frequently reported, we advise interpreting these values cautiously due to the absence of reports on the reference standard for infection used.

### Towards routine implementation of serological diagnosis to guide STH prevention and control programs

While a substantial number of assays are employed in epidemiological research, the current state of serological diagnosis for guiding STH prevention and control programs is limited. Only two assays developed for pigs are used to inform deworming strategies in these populations, inferred from their product information that provides guidelines for sampling design (diagnosis of pig herd infestation based on periodic sampling of 10 animals) and interpretation criteria. For human diagnostics, there is a noticeable gap, as none of the existing assays have been integrated into routine diagnostics within MDA programs, or was even validated on a large scale. While it is indeed debatable whether reports of routine implementation to guide prevention and control in veterinary medicine would be present in the scientific literature, the situation is different for human MDA programs. Clear examples exist, such as the prevalence studies conducted by Leta et al. (2020) in Ethiopia [96], Tchuenté et al. (2012) in Cameroon [97], Ibikounlé et al. (2018) in Benin [98], and Koroma et al. (2010) in Sierra Leone [99]. These studies, which all employed the Kato-Katz thick smear method, have been instrumental in mapping the prevalence of STHs in these regions, and provide the necessary tools for their Ministries of Health to plan national control programs. Specifically concerning large-scale validation of assays, the most extensive sample set reported in the included records was less than 7,000 individuals, sourced cross-sectionally from a single country [45]. For a broader perspective, large-scale validation efforts comparable in size to the Geshiyaro Project (annual collection of over 6,000 samples) [100] or Deworm3 (longitudinal sampling of 500–1,000 individuals across 40 clusters) [101,102] are essential. Moreover, since guiding STH prevention and control is the ultimate purpose, this suggests that validation should also encompass progress towards these objectives, providing guidelines for sampling design and program decisions, like the WHO guidelines currently used for Kato-Katz thick smear [103].

This review identifies important gaps that might be contributing to the present limited state of serological assay implementation in human MDA programs. First of all, for the transition from Stage 1 (assay development) to Stage 2 (cut-off establishment), meticulously characterized sample sets play a key role. In veterinary medicine, obtaining these samples via animal trials is feasible, whereas biobanks with extensively characterized human samples are not readily available. Access to such biobanks has the potential to significantly accelerate the validation of new assays. Of particular interest are sample sets that not only focus on STHs but also comprehensively identify and thoroughly document other NTDs and non-NTDs infections. Additionally, it will be important that these samples are made accessible to research groups worldwide, facilitating meaningful head-to-head comparisons between studies and assays.

To make the transition from Stage 2 (cut-off establishment) to Stage 3 (research use), our assessments revealed that it will be important to standardize reporting practices, including (i) precise sample set descriptions, (ii) the chosen reference standard for assessing STH infection, and the statistical methodologies employed in both (iii) defining cut-off values and (iv) evaluating the diagnostic performance. This might eventually streamline research, which in turn will be useful in refining and optimizing serological assays for routine implementation. In this context, the establishment of comprehensive assay inventories as currently undertaken by FIND (https://www.finddx.org/ntds/test-directory) can play a key role. Additionally, the present scoping review can assist researchers by preventing replication in experiments. Our current focus was not on detailed study quality assessment or outcome synthesis, as these tasks are beyond the scope of a scoping review. However, this study can be a foundational step for a future systematic review that focusses on providing a critical analysis of the research conducted with the aim to draw conclusions regarding the quality of the existing assays.

As mentioned earlier, the transition from research use (Stage 3) to routine implementation (Stage 4) requires large-scale performance validation that also includes establishment of guidelines for program decision-making. First, it is important to acknowledge that the primary aim of research groups behind developing assays might not always be to facilitate program decision-making. Second, the concept of ‘validation’ depends entirely on the question ‘what are the necessary characteristics of a useful serodiagnostic test’, which in turn relies completely on a thorough understanding of the specific outcomes we aim to measure. In the context of animal production systems, where animals have a more limited lifespan, simply identifying prior infection levels might be sufficient for enhancing future prevention and control strategies. In contrast, the implementation in human MDA programs is much more complex. Essential factors to consider include whether the assay is intended for mapping, M&E or post-program surveillance, and the way the results are interpreted (e.g., determining prevalence, analysing quantitative results, and assessing recurrence of the disease). These questions go beyond the difference between measuring exposure by serology *vs*. detecting patent infection through Kato-Katz thick smear, delving deeper into the strategic use of serological data to guide informed decision-making processes. Furthermore, if we consider blood samples as a way forward for more integrated M&E of NTD programs and an opportunity to integrate NTDs into existing public health surveillance platforms, a significant challenge lies in the validation of multiplex assays. One of the questions is whether these assays should conform to the specific TPPs for each disease, or if there is room for flexibility in terms of diagnostic sensitivity and specificity, particularly when examining larger populations or adopting multi-step testing approaches (e.g., serodiagnostic assay with high sensitivity followed by a test with high specificity). Furthermore, in the context of STH diagnostics, a pan-helminth test capable of detecting all three species (*Ascaris*, *Trichuris* and hookworms), therefore however not meeting the TPPs, might present a potential for post-program surveillance. In summary, addressing many of these core questions in both the STH and wider NTD community will be crucial for progressing towards the routine implementation of serology in NTD prevention and control.

## Conclusion

We identified 85 relevant literature records spanning over 50 years, with a notable increased interest in serological assay development in recent years. Most of the research efforts concentrated on diagnosing *Ascaris* infections in both humans and pigs, primarily using ELISA and western blot technologies. Almost all records targeted antibodies as analytes, employing proteins and peptides as analyte detection agents. Approximately 60% of sample sets described pertained to human samples. No commercially available tests for *Trichuris* or hookworms were identified, while for *Ascaris*, there are at least seven different ELISAs on the market. While a substantial number of assays are employed in epidemiological research, the current state of serological diagnosis for guiding STH prevention and control programs is limited. Only two assays designed for pigs are used to inform efficient deworming practices in pig populations. This scoping review identified factors that potentially contribute to the present limited implementation of serological assays in human MDA programs, despite their demonstrated potential in veterinary medicine. The challenges include the lack of well-documented human sample sets and the absence of reporting standards and assay inventories. Additionally, it highlighted numerous critical questions regarding the strategic use of serological data to facilitate informed decision-making. Addressing these challenges and questions is essential for enhancing the integration of serological approaches into NTD prevention and control efforts.

## Supporting information

S1 Info Preferred Reporting Items for Systematic Reviews and Meta-Analyses extension for Scoping Reviews (PRISMA-ScR) checklist. (PDF)

S2 Info Detailed search strategies in Ovid MEDLINE, Embase, Cochrane Library, Google and Espacenet. Our search strategy comprised two distinct components: (i) a literature search for published records and (ii) an exploration of commercially available assays and diagnostic services, and patents that either resulted in or could potentially result in commercial assays.
(PDF)

S3 Info Comprehensive summary of the 85 literature records included in this scoping review. The table includes (i) the year of publication, (ii) details about authors and research group, (iii) the advancement stage according to criteria described in **Table 3**, (iv) the test species, (v) details on the study population, (vi) the analyte detection agent used, (vii) the assay technology principle applied, (viii) the target analyte detected, (ix) the source of the assay, and (x) specific assay parameters. We have clustered these details on an STH species level. For assays targeting *Ascaris*, the list also includes (xi) the target disease (intestinal parasitosis or LMS). The level of detail stated in the table is limited to that provided in the articles. In the context of *Ascaris*, when not specified, we are referring to intestinal parasitosis (e.g., for positive/negative individuals).
(XLSX)

S4 Info Characteristics of the assays used for serodiagnosis of STHs considering the evolving landscape of research over time. The number of records by technology principle (page 1), target analyte detected (page 2) and analyte detection agent used (page 3). Records are presented in historical order by year of publication. For target analyte and analyte detection agent, we have clustered the results on an STH species level. In cases where a record used multiple options, the records was included in the count of all relevant options. FAT: fluorescent antibody test, ELISA: enzyme-linked immunosorbent assay, MFI: multiplex flow immunoassay, qSAT: quantitative suspension array technology, SDS-PAGE: sodium dodecyl sulfate polyacrylamide gel electrophoresis, REIA: reverse enzyme immunoassay, RIA: radioimmunoassay, Ab: antibody, Ig: immunoglobulin, IC: immunecomplex, ES: excretory/secretory, HW: hookworm, STH: soil-transmitted helminth.
(PDF)

S5 Info Summary of all the references used to create Fig 5. References are presented in a table consistent with Fig 5 itself.
(PDF)

S6 Info Overview of the patent documents related to serological diagnosis for soil-transmitted helminthiases. Records were retrieved by an Espacenet patent search. NA: information not available.
(PDF)

## References

[1] World Health Organization. Schistosomiasis and soil-transmitted helminthiases: progress report, 2021. Weekly epidemiological record. 2022;97(48):621–632.

[2] World Health Organization. Ending the neglect to attain the Sustainable Development Goals: a road map for neglected tropical diseases 2021–2030. Geneva; 2020. Licence: CC BY-NC-SA 3.0 IGO.

[3] Crossing the billion. Lymphatic filariasis, onchocerciasis, schistosomiasis, soil-transmitted helminthiases and trachoma: preventive chemotherapy for neglected tropical diseases. Geneva: World Health Organization; 2017. Licence: CC BY-NC-SA 3.0 IGO.

[4] Safety in administering medicines for neglected tropical diseases. Geneva: World Health Organization; 2021. Licence: CC BY-NC-SA 3.0 IGO.

[5] Report of the first meeting of the WHO Diagnostic Technical Advisory Group for Neglected Tropical Diseases, Geneva, Switzerland, 30–31 October 2019. Geneva: World Health Organization; 2020. Licence: CC BY-NC-SA 3.0 IGO.

[6] Lammie P SA, Solomon A, Secor E, Peeling R. Diagnostic needs for NTD programs. In: Institute of Medicine (US) Forum on Microbial Threats The Causes and Impacts of Neglected Tropical and Zoonotic Diseases: Opportunities for Integrated Intervention Strategies. Washington (DC): National Academies Press (US); 2011. A14. Available from: https://www.ncbi.nlm.nih.gov/books/NBK62529/. 21977543

[7] Solomon AW, Engels D, Bailey RL, Blake IM, Brooker S, Chen J-X, et al. A Diagnostics Platform for the Integrated Mapping, Monitoring, and Surveillance of Neglected Tropical Diseases: Rationale and Target Product Profiles. Plos Neglect Trop D. 2012;6(7):e1746. doi: 10.1371/journal.pntd.0001746 22860146 PMC3409112

[8] Souza AA, Ducker C, Argaw D, King JD, Solomon AW, Biamonte MA, et al. Diagnostics and the neglected tropical diseases roadmap: setting the agenda for 2030. T Roy Soc Trop Med H. 2020;115(2):129–35. doi: 10.1093/trstmh/traa118 33169166 PMC7842105

[9] World Health Organization. Diagnostic test for surveillance of lymphatic filariasis: target product profile. Geneva. Licence: CC BY-NC-SA 3.0 IGO. 2021.

[10] World Health Organization. Onchocerciasis: diagnostic target product profile to support preventive chemotherapy. Licence: CC BY-NC-SA 3.0 IGO. 2021.

[11] Target product profile for scabies to start and stop mass drug administration. Geneva: World Health Organization; 2022. Licence: CC BY-NC-SA 3.0 IGO.

[12] World Health Organization. Diagnostic target product profiles for monitoring, evaluation and surveillance of schistosomiasis control programmes. Geneva. Licence: CC BY-NC-SA 3.0 IGO. 2021.

[13] World Health Organization. Diagnostic target product profiles for monitoring and evaluation of soil-transmitted helminth control programs. 2021. Licence: CC BY-NC-SA 3.0 IGO.

[14] World Health Organization. Call for public consultation –Target Product Profiles (TPP) for trachoma surveillance 2023 [cited 2023 14/09]. Available from: https://www.who.int/news-room/articles-detail/call-for-public-consultation—target-product-profiles-(tpp)-for-trachoma-surveillance.

[15] Wiens KE, Jauregui B, Arnold BF, Banke K, Wade D, Hayford K, et al. Building an integrated serosurveillance platform to inform public health interventions: Insights from an experts’ meeting on serum biomarkers. PLOS Neglected Tropical Diseases. 2022;16(10):e0010657. doi: 10.1371/journal.pntd.0010657 36201428 PMC9536637

[16] Metcalf CJE, Farrar J, Cutts FT, Basta NE, Graham AL, Lessler J, et al. Use of serological surveys to generate key insights into the changing global landscape of infectious disease. The Lancet. 2016;388(10045):728–30. doi: 10.1016/S0140-6736(16)30164-7 27059886 PMC5678936

[17] World Health Organization. Guidelines for Stopping Mass Drug Administration and Verifying Elimination of Human Onchocerciasis: Criteria and Procedures. WHO Guidelines Approved by the Guidelines Review Committee. Geneva: World Health Organization.; 2016. 26913317

[18] World Health Organization. Monitoring and epidemiological assessment of mass drug administration in the global programme to eliminate lymphatic filariasis: a manual for national elimination programmes. Geneva: World Health Organization; 2011.

[19] Raw C, Traub RJ, Zendejas-Heredia PA, Stevenson M, Wiethoelter A. A systematic review and meta-analysis of human and zoonotic dog soil-transmitted helminth infections in Australian Indigenous communities. PLOS Neglected Tropical Diseases. 2022;16(10):e0010895. doi: 10.1371/journal.pntd.0010895 36279298 PMC9632820

[20] Zheng Y, Xie Y, Geldhof P, Vlaminck J, Ma G, Gasser RB, et al. High anti-Ascaris seroprevalence in fattening pigs in Sichuan, China, calls for improved management strategies. Parasites & vectors. 2020;13(1):60. doi: 10.1186/s13071-020-3935-4 32051006 PMC7017502

[21] Munn Z, Peters MDJ, Stern C, Tufanaru C, McArthur A, Aromataris E. Systematic review or scoping review? Guidance for authors when choosing between a systematic or scoping review approach. BMC Medical Research Methodology. 2018;18(1):143. doi: 10.1186/s12874-018-0611-x 30453902 PMC6245623

[22] Peters MDJ, Marnie C, Tricco AC, Pollock D, Munn Z, Alexander L, et al. Updated methodological guidance for the conduct of scoping reviews. JBI Evidence Synthesis. 2020;18(10). doi: 10.11124/JBIES-20-00167 33038124

[23] Tricco AC, Lillie E, Zarin W, O’Brien KK, Colquhoun H, Levac D, et al. PRISMA Extension for Scoping Reviews (PRISMA-ScR): Checklist and Explanation. Annals of Internal Medicine. 2018;169(7):467–73. doi: 10.7326/M18-0850 30178033

[24] The EndNote Team. EndNote. EndNote X9 ed. Philadelphia, PA: Clarivate; 2013.

[25] Else KJ, Keiser J, Holland CV, Grencis RK, Sattelle DB, Fujiwara RT, et al. Whipworm and roundworm infections. Nat Rev Dis Primers. 2020;6(1):44. Epub 2020/05/30. doi: 10.1038/s41572-020-0171-3 . 32467581

[26] Yoshida A, Hombu A, Wang Z, Maruyama H. Larva migrans syndrome caused by Toxocara and Ascaris roundworm infections in Japanese patients. Eur J Clin Microbiol Infect Dis. 2016;35(9):1521–9. Epub 2016/06/09. doi: 10.1007/s10096-016-2693-x ; PubMed Central PMCID: PMC4982883. 27272122 PMC4982883

[27] R Core Team (2019). R: A language and environment for statistical computing. R Foundation for Statis- tical Computing, Vienna, Austria. Available from: http://R-project.org

[28] Ganguly NK, Mahajan RC, Sehgal R, Shetty P, Dilawari JB. Role of specific immunoglobulin E to excretory-secretory antigen in diagnosis and prognosis of hookworm infection. Journal of clinical microbiology. 1988;26(4):739–42. doi: 10.1128/jcm.26.4.739-742.1988 3284900 PMC266433

[29] Lopes CA, de Faria LS, de Sousa JEN, Borges IP, Ribeiro RP, Bueno LL, et al. Anti-Ascaris suum immunoglobulin Y as a novel biotechnological tool for the diagnosis of human ascariasis. Journal of helminthology. 2019;94:e71. doi: 10.1017/S0022149X19000701 31409433

[30] Maruyama H, Noda S, Choi W-Y, Ohta N, Nawa Y. Fine binding specificities to Ascaris suum and Ascaris lumbricoides antigens of the sera from patients of probable visceral larva migrans due to Ascaris suum. Parasitology International. 1997;46(3):181–8. 10.1016/S1383-5769(97)00025-1.

[31] Welch JS, Dobson C. Immunodiagnosis of parasitic zoonoses: comparative efficacy of three immunofluorescence tests using antigens purified by affinity chromatography. Transactions of the Royal Society of Tropical Medicine and Hygiene. 1978;72(3):282–8. doi: 10.1016/0035-9203(78)90209-2 354114

[32] Welch JS, Dobson C, Chopra S. Immunodiagnosis of Entamoeba histolytica and Ascaris lumbricoides infections in Caucasian and Aboriginal Australians. Transactions of the Royal Society of Tropical Medicine and Hygiene. 1986;80(2):240–7. doi: 10.1016/0035-9203(86)90024-6 2878514

[33] Tanaka K, Kawamura H, Tohgi N, Tsuji M, Miyachi Y, Miyoshi A. The measurement of Ascaris suum protein by radioimmunoassay in sera from patients with helminthiasis and with gastrointestinal diseases. Parasitology. 1983;86 (Pt 2):291–300. doi: 10.1017/s0031182000050459 6682964

[34] Patterson R, Huntley CC, Roberts M, Irons JS. Visceral larva migrans: Immunoglobulins, precipitating antibodies and detection of IgG and IgM antibodies against Ascaris antigen. The American journal of tropical medicine and hygiene. 1975;24(3):465–70. doi: 10.4269/ajtmh.1975.24.465 1155690

[35] Yoshihara S, Nakagawa M, Suda H. Detection of complement fixation antibody against Ascaris suum antigen in pigs with white spots in the liver. Nihon Juigaku Zasshi. 1987;49(3):559–61. Epub 1987/06/01. doi: 10.1292/jvms1939.49.559 . 3613356

[36] Yoshihara S, Oya T, Furuya T, Goto N. Use of body fluid of adult female Ascaris suum as an antigen in the enzyme-linked immunosorbent assay (ELISA) for diagnosis of swine ascariosis. Journal of helminthology. 1993;67(4):279–86. doi: 10.1017/s0022149x00013274 8132972

[37] Takashima M, Ohmi H, Watanabe T, Okamoto K, Kanoe M, Nagai S. Attempts to separate female Ascaris suum antigen and to investigate its partial characterization. Veterinary journal (London, England: 1997). 2003;165(2):164–8. doi: 10.1016/s1090-0233(02)00166-1 12573606

[38] Njenga SM, Kanyi HM, Arnold BF, Matendechero SH, Onsongo JK, Won KY, et al. Integrated Cross-Sectional Multiplex Serosurveillance of IgG Antibody Responses to Parasitic Diseases and Vaccines in Coastal Kenya. The American journal of tropical medicine and hygiene. 2020;102(1):164–76. doi: 10.4269/ajtmh.19-0365 31769388 PMC6947807

[39] Vlaminck J, Lagatie O, Dana D, Mekonnen Z, Geldhof P, Levecke B, et al. Identification of antigenic linear peptides in the soil-transmitted helminth and Schistosoma mansoni proteome. PLoS Negl Trop Dis. 2021;15(4):e0009369. Epub 2021/04/29. doi: 10.1371/journal.pntd.0009369 . 33909616 PMC8081252

[40] Vengesai A, Naicker T, Midzi H, Kasambala M, Mduluza-Jokonya TL, Rusakaniko S, et al. Multiplex peptide microarray profiling of antibody reactivity against neglected tropical diseases derived B-cell epitopes for serodiagnosis in Zimbabwe. PloS one. 2022;17(7):e0271916. doi: 10.1371/journal.pone.0271916 35867689 PMC9307155

[41] Santano R, Rubio R, Grau-Pujol B, Escola V, Muchisse O, Cuamba I, et al. Plasmodium falciparum and Helminth Coinfections Increase IgE and Parasite-Specific IgG Responses. Microbiology spectrum. 2021;9(3):e0110921. doi: 10.1128/Spectrum.01109-21 34878303 PMC8653825

[42] Santano R, Rubio R, Grau-Pujol B, Escola V, Muchisse O, Cuamba I, et al. Evaluation of antibody serology to determine current helminth and Plasmodium falciparum infections in a co-endemic area in Southern Mozambique. PLoS neglected tropical diseases. 2022;16(6):e0010138. doi: 10.1371/journal.pntd.0010138 35727821 PMC9212154

[43] Dana D, Vlaminck J, Ayana M, Tadege B, Mekonnen Z, Geldhof P, et al. Evaluation of copromicroscopy and serology to measure the exposure to Ascaris infections across age groups and to assess the impact of 3 years of biannual mass drug administration in Jimma Town, Ethiopia. PLoS neglected tropical diseases. 2020;14(4):e0008037. doi: 10.1371/journal.pntd.0008037 32282815 PMC7179930

[44] Dana D, Roose S, Vlaminck J, Ayana M, Mekonnen Z, Geldhof P, et al. Longitudinal assessment of the exposure to Ascaris lumbricoides through copromicroscopy and serology in school children from Jimma Town, Ethiopia. PLoS neglected tropical diseases. 2022;16(1):e0010131. doi: 10.1371/journal.pntd.0010131 35041666 PMC8797258

[45] Roose S, Leta GT, Vlaminck J, Getachew B, Mekete K, Peelaers I, et al. Comparison of coproprevalence and seroprevalence to guide decision-making in national soil-transmitted helminthiasis control programs: Ethiopia as a case study. PLoS Negl Trop Dis. 2022;16(10):e0010824. Epub 2022/10/06. doi: 10.1371/journal.pntd.0010824 ; PubMed Central PMCID: PMC9534397. 36197895 PMC9534397

[46] Santra A, Bhattacharya T, Chowdhury A, Ghosh A, Ghosh N, Chatterjee BP, et al. Serodiagnosis of ascariasis with specific IgG4 antibody and its use in an epidemiological study. Transactions of the Royal Society of Tropical Medicine and Hygiene. 2001;95(3):289–92. doi: 10.1016/s0035-9203(01)90236-6 11490999

[47] Vlaminck J, Supali T, Geldhof P, Hokke CH, Fischer PU, Weil GJ. Community Rates of IgG4 Antibodies to Ascaris Haemoglobin Reflect Changes in Community Egg Loads Following Mass Drug Administration. PLoS neglected tropical diseases. 2016;10(3):e0004532. doi: 10.1371/journal.pntd.0004532 26991326 PMC4798312

[48] Cruz K, Marcilla A, Kelly P, Vandenplas M, Osuna A, Trelis M. Trichuris trichiura egg extract proteome reveals potential diagnostic targets and immunomodulators. PLoS neglected tropical diseases. 2021;15(3):e0009221. doi: 10.1371/journal.pntd.0009221 33760829 PMC8021180

[49] Pritchard DI, Walsh EA. The specificity of the human IgE response to Necator americanus. Parasite Immunology. 1995;17(11):605–7. doi: 10.1111/j.1365-3024.1995.tb01005.x 8817608

[50] Palmer. IgG4 responses to antigens of adult Necator americanus: potential for use in large-scale epidemiological studies. 1996. Bulletin of the World Health Organization, 1996, 74 (4): 381–386 PMC2486892 8823959

[51] Loukas A, Opdebeeck J, Croese J, Prociv P. Immunoglobulin G subclass antibodies against excretory/secretory antigens of Ancylostoma caninum in human enteric infections. The American journal of tropical medicine and hygiene. 1996;54(6):672–6. doi: 10.4269/ajtmh.1996.54.672 8686791

[52] DuVall AS, Fairley JK, Sutherland L, Bustinduy AL, Mungai PL, Muchiri EM, et al. Development of a specimen-sparing multichannel bead assay to detect antiparasite IgG4 for the diagnosis of Schistosoma and Wuchereria infections on the coast of Kenya. Am J Trop Med Hyg. 2014;90(4):63845. Epub 2014/02/12. doi: 10.4269/ajtmh.13-0292 ; PubMed Central PMCID: PMC3973507. 24515945 PMC3973507

[53] Logan J, Pearson MS, Manda SS, Choi Y-J, Field M, Eichenberger RM, et al. Comprehensive analysis of the secreted proteome of adult Necator americanus hookworms. PLoS neglected tropical diseases. 2020;14(5):e0008237. 10.1371/journal.pntd.0008237. 32453752 PMC7274458

[54] Souza DCd, de Faria LS, Sousa JENd, Lopes CdA, Ribeiro VdS, da Silva VJ, et al. Use of polyclonal IgY antibodies to detect serum immune complexes in patients with active hookworm infection. Parasitology. 2020;147(6):715–20. doi: 10.1017/S0031182020000220 32051048 PMC10317617

[55] Bøgh HO, Eriksen L, Lawson LG, Lind P. Evaluation of an enzyme-linked immunosorbent assay and a histamine release test system for the detection of pigs naturally infected with Ascaris suum. Preventive Veterinary Medicine. 1994;21(3):201–14. 10.1016/0167-5877.

[56] Lind P, Eriksen L, Nansen P, Nilsson O, Roepstorff A. Response to repeated inoculations with Ascaris suum eggs in pigs during the fattening period. II. Specificf IgA, IgG, and IgM antibodies determined by enzyme-linked immunosorbent assay. Parasitology Research. 1993;79(3):240–4. 8493248 10.1007/BF00931899

[57] Frontera E, Serrano F, Reina D, Alcaide M, Sanchez-Lopez J, Navarrete I. Serological responses to Ascaris suum adult worm antigens in Iberian finisher pigs. Journal of helminthology. 2003;77(2):167–72. doi: 10.1079/JOH2002163 12756071

[58] Pritchard DI, Quinnell RJ, Slater AF, McKean PG, Dale DD, Raiko A, et al. Epidemiology and immunology of Necator americanus infection in a community in Papua New Guinea: humoral responses to excretory-secretory and cuticular collagen antigens. Parasitology. 1990;100 Pt 2:317–26. Epub 1990/04/01. doi: 10.1017/s0031182000061333 . 2345664

[59] Vlaminck J, Nejsum P, Vangroenweghe F, Thamsborg SM, Vercruysse J, Geldhof P. Evaluation of a serodiagnostic test using Ascaris suum haemoglobin for the detection of roundworm infections in pig populations. Veterinary parasitology. 2012;189(2–4):267–73. doi: 10.1016/j.vetpar.2012.04.024 22560331

[60] Vlaminck J, Dusseldorf S, Heres L, Geldhof P. Serological examination of fattening pigs reveals associations between Ascaris suum, lung pathogens and technical performance parameters. Veterinary parasitology. 2015;210(3–4):151–8. doi: 10.1016/j.vetpar.2015.04.012 25952722

[61] Bundy DA, Lillywhite JE, Didier JM, Simmons I, Bianco AE. Age-dependency of infection status and serum antibody levels in human whipworm (Trichuris trichiura) infection. Parasite immunology. 1991;13(6):629–38. doi: 10.1111/j.1365-3024.1991.tb00558.x 1811214

[62] Lillywhite JE, Bundy DA, Didier JM, Cooper ES, Bianco AE. Humoral immune responses in human infection with the whipworm Trichuris trichiura. Parasite Immunol. 1991;13(5):491–507. Epub 1991/09/01. doi: 10.1111/j.1365-3024.1991.tb00546.x . 1956697

[63] Needham CS, Bundy DAP, Lillywhite JE, Didier JM, Simmons I, Bianco AE. The relationship between Trichuris trichiura transmission intensity and the age-profiles of parasite-specific antibody isotypes in two endemic communities. Parasitology. 1992;105(2):273–83. Epub 2009/04/06. doi: 10.1017/s0031182000074205 1454425

[64] Needham CS, Lillywhite JE, Didier JM, Bianco AE, Bundy DAP. Age-dependency of serum isotype responses and antigen recognition in human whipworm (Trichuris trichiura) infection. Parasite Immunology. 1993;15(12):683–92. doi: 10.1111/j.1365-3024.1993.tb00583.x 7533282

[65] Needham CS, Lillywhite JE, Didier JM, Bianco AE, Bundy DAP. Temporal changes in Trichuris trichiura infection intensity and serum isotype responses in children. Parasitology. 1994;109(2):197–200. Epub 2009/04/06. doi: 10.1017/s0031182000076307 8084665

[66] Needham CS, Lillywhite JE, Didier JM, Bianco AT, Bundy DAP. Comparison of age-dependent antigen recognition in two communities with high and low Trichuris trichiura transmission. Acta Tropica. 1994;58(2):87–98. doi: 10.1016/0001-706x(94)90048-5 7887344

[67] Needham CS, Lillywhite JE, Didier JM, Bundy DAP. Serum isotype responses after treatment of human trichuriasis. Transactions of The Royal Society of Tropical Medicine and Hygiene. 1994;88(3):354–5. doi: 10.1016/0035-9203(94)90114-7 7974688

[68] Lillywhite JE, Cooper ES, Needham CS, Venugopal S, Bundy DAP, Bianco AE. Identification and characterization of excreted/secreted products of Trichuris trichiura. Parasite Immunology. 1995;17(1):47–54. doi: 10.1111/j.1365-3024.1995.tb00965.x 7731735

[69] Malafiej E, Spiewak E. Serological investigation in children infected with Ascaris lumbricoides. Wiadomosci parazytologiczne. 2001;47(4):585–90. 16886394

[70] Pinelli E, Herremans T, Harms MG, Hoek D, Kortbeek LM. Toxocara and Ascaris seropositivity among patients suspected of visceral and ocular larva migrans in the Netherlands: trends from 1998 to 2009. European journal of clinical microbiology & infectious diseases: official publication of the European Society of Clinical Microbiology. 2011;30(7):873–9. doi: 10.1007/s10096-011-1170-9 21365288

[71] Schneider R, Obwaller A, Auer H. Immunoblot for the detection of Ascaris suum-specific antibodies in patients with visceral larva migrans (VLM) syndrome. Parasitology research. 2015;114(1):305–10. doi: 10.1007/s00436-014-4196-y 25367210

[72] Schneider R, Auer H. Incidence of Ascaris suum-specific antibodies in Austrian patients with suspected larva migrans visceralis (VLM) syndrome. Parasitology research. 2016;115(3):1213–9. doi: 10.1007/s00436-015-4857-5 26637313

[73] Mughini-Gras L, Harms M, van Pelt W, Pinelli E, Kortbeek T. Seroepidemiology of human Toxocara and Ascaris infections in the Netherlands. Parasitology research. 2016;115(10):3779–94. doi: 10.1007/s00436-016-5139-6 27234034

[74] Lassen B, Janson M, Viltrop A, Neare K, Hutt P, Golovljova I, et al. Serological Evidence of Exposure to Globally Relevant Zoonotic Parasites in the Estonian Population. PloS one. 2016;11(10):e0164142. doi: 10.1371/journal.pone.0164142 27723790 PMC5056716

[75] Taus K, Schmoll F, El-Khatib Z, Auer H, Holzmann H, Aberle S, et al. Occupational swine exposure and Hepatitis E virus, Leptospira, Ascaris suum seropositivity and MRSA colonization in Austrian veterinarians, 2017-2018-A cross-sectional study. Zoonoses and public health. 2019;66(7):842–51. doi: 10.1111/zph.12633 31419070 PMC6851874

[76] Chatterjee BP, Santra A, Karmakar PR, Mazumder DN. Evaluation of IgG4 response in ascariasis by ELISA for serodiagnosis. Tropical medicine & international health: TM & IH. 1996;1(5):633–9. doi: 10.1111/j.1365-3156.1996.tb00088.x 8911447

[77] Vlaminck J, Masure D, Wang T, Nejsum P, Hokke CH, Geldhof P. A Phosphorylcholine-Containing Glycolipid-like Antigen Present on the Surface of Infective Stage Larvae of Ascaris spp. Is a Major Antibody Target in Infected Pigs and Humans. PLoS neglected tropical diseases. 2016;10(12):e0005166. doi: 10.1371/journal.pntd.0005166 27906979 PMC5131908

[78] Shetty P, Dilawari JB, Virk KJ, Sehgal R, Ganguly NK, Mahajan RC. Enzyme-linked immunosorbent assay using excretory/secretory and somatic antigens as a diagnostic test for human hookworm infection. Transactions of the Royal Society of Tropical Medicine and Hygiene. 1988;82(5):736–8. doi: 10.1016/0035-9203(88)90220-9 3252592

[79] Vandekerckhove E, Vlaminck J, Geldhof P. Evaluation of serology to measure exposure of piglets to Ascaris suum during the nursery phase. Veterinary parasitology. 2017;246:82–7. doi: 10.1016/j.vetpar.2017.09.008 28969785

[80] Farah IO, Kariuki TM, King CL, Hau J. An overview of animal models in experimental schistosomiasis and refinements in the use of non-human primates. Lab Anim. 2001;35(3):205–12. Epub 2001/07/21. doi: 10.1258/0023677011911570 . 11463066

[81] Shepherd C, Wangchuk P, Loukas A. Of dogs and hookworms: man’s best friend and his parasites as a model for translational biomedical research. Parasit Vectors. 2018;11(1):59. Epub 2018/01/27. doi: 10.1186/s13071-018-2621-2 ; PubMed Central PMCID: PMC5785905. 29370855 PMC5785905

[82] Kringel H, Thamsborg SM, Petersen HH, Göring HHH, Skallerup P, Nejsum P. Serum antibody responses in pigs trickle-infected with Ascaris and Trichuris: Heritabilities and associations with parasitological findings. Veterinary Parasitology. 2015;211(3):306–11. doi: 10.1016/j.vetpar.2015.06.008 26095952

[83] Fujiwara RT, Geiger SM, Bethony J, Mendez S. Comparative immunology of human and animal models of hookworm infection. Parasite Immunol. 2006;28(7):285–93. Epub 2006/07/18. doi: 10.1111/j.1365-3024.2006.00821.x . 16842265

[84] Lynch NR, Hagel I, Vargas V, Rotundo A, Varela MC, Di Prisco MC, et al. Comparable seropositivity for ascariasis and toxocariasis in tropical slum children. Parasitology research. 1993;79(7):547–50. doi: 10.1007/BF00932238 8278336

[85] Hill DE, Romanowski RD, Urban JF, Jr. A Trichuris specific diagnostic antigen from culture fluids of Trichuris suis adult worms. Veterinary parasitology. 1997;68(1–2):91–102. doi: 10.1016/s0304-4017(96)01055-2 9066055

[86] Fortunato S, Castagna B, Monteleone MR, Pierro R, Cringoli G, Bruschi F. Parasite prevalence in a village in Burkina Faso: the contribution of new techniques. Journal of infection in developing countries. 2014;8(5):670–5. doi: 10.3855/jidc.3660 24820474

[87] Guo F, Forde MS, Werre SR, Krecek RC, Zhu G. Seroprevalence of five parasitic pathogens in pregnant women in ten Caribbean countries. Parasitology research. 2017;116(1):347–58. doi: 10.1007/s00436-016-5297-6 27778108

[88] van Knapen F, van Leusden J, Polderman AM, Franchimont JH. Visceral larva migrans: examinations by means of enzyme-linked immunosorbent assay of human sera for antibodies to excretory-secretory antigens of the second-stage larvae of Toxocara canis. Z Parasitenkd. 1983;69(1):113–8. Epub 1983/01/01. doi: 10.1007/BF00934015 . 6837095

[89] Martinez-Perez JM, Vandekerckhove E, Vlaminck J, Geldhof P, Martinez-Valladares M. Serological detection of Ascaris suum at fattening pig farms is linked with performance and management indices. Veterinary parasitology. 2017;248:33–8. doi: 10.1016/j.vetpar.2017.10.009 29173538

[90] Lassen B, Geldhof P, Halli O, Vlaminck J, Oliviero C, Orro T, et al. Anti-Ascaris suum IgG antibodies in fattening pigs with different respiratory conditions. Veterinary parasitology. 2019;265:85–90. doi: 10.1016/j.vetpar.2018.12.005 30638525

[91] Joachim A, Winkler C, Ruczizka U, Ladinig A, Koch M, Tichy A, et al. Comparison of different detection methods for Ascaris suum infection on Austrian swine farms. Porcine health management. 2021;7(1):57. doi: 10.1186/s40813-021-00236-9 34666834 PMC8524899

[92] Delsart M, Fablet C, Rose N, Reperant J-M, Blaga R, Dufour B, et al. Descriptive epidemiology of the main internal parasites on alternative pig farms in france. The Journal of parasitology. 2022;108(4):306–21. doi: 10.1645/21-126 35877156

[93] Tassis P, Symeonidou I, Gelasakis AI, Kargaridis M, Aretis G, Arsenopoulos KV, et al. Serological Assessment of Ascaris suum Exposure in Greek Pig Farms and Associated Risk Factors Including Lawsonia intracellularis. Pathogens (Basel, Switzerland). 2022;11(9). doi: 10.3390/pathogens11090959 36145391 PMC9503870

[94] Lassen B, Oliviero C, Orro T, Jukola E, Laurila T, Haimi-Hakala M, et al. Effect of fenbendazole in water on pigs infected with Ascaris suum in finishing pigs under field conditions. Veterinary parasitology. 2017;237:1–7. doi: 10.1016/j.vetpar.2017.03.005 28285891

[95] Chehayeb JF, Robertson AP, Martin RJ, Geary TG. Proteomic analysis of adult Ascaris suum fluid compartments and secretory products. PLoS Negl Trop Dis. 2014;8(6):e2939. Epub 2014/06/06. doi: 10.1371/journal.pntd.0002939 ; PubMed Central PMCID: PMC4046973. 24901219 PMC4046973

[96] Leta GT, Mekete K, Wuletaw Y, Gebretsadik A, Sime H, Mekasha S, et al. National mapping of soil-transmitted helminth and schistosome infections in Ethiopia. Parasite Vector. 2020;13(1):437. doi: 10.1186/s13071-020-04317-6 32873333 PMC7466696

[97] Tchuem Tchuenté LA, Kamwa Ngassam RI, Sumo L, Ngassam P, Dongmo Noumedem C, Nzu DD, et al. Mapping of schistosomiasis and soil-transmitted helminthiasis in the regions of centre, East and West Cameroon. PLoS Negl Trop Dis. 2012;6(3):e1553. Epub 2012/03/14. doi: 10.1371/journal.pntd.0001553 ; PubMed Central PMCID: PMC3295801. 22413029 PMC3295801

[98] Ibikounlé M, Onzo-Aboki A, Doritchamou J, Tougoué JJ, Boko PM, Savassi BS, et al. Results of the first mapping of soil-transmitted helminths in Benin: Evidence of countrywide hookworm predominance. PLoS Negl Trop Dis. 2018;12(3):e0006241. Epub 2018/03/02. doi: 10.1371/journal.pntd.0006241 ; PubMed Central PMCID: PMC5849360. 29494579 PMC5849360

[99] Koroma JB, Peterson J, Gbakima AA, Nylander FE, Sahr F, Soares Magalhães RJ, et al. Geographical distribution of intestinal schistosomiasis and soil-transmitted helminthiasis and preventive chemotherapy strategies in Sierra Leone. PLoS Negl Trop Dis. 2010;4(11):e891. Epub 2010/12/03. doi: 10.1371/journal.pntd.0000891 ; PubMed Central PMCID: PMC2990690. 21124881 PMC2990690

[100] Mekete K, Ower A, Dunn J, Sime H, Tadesse G, Abate E, et al. The Geshiyaro Project: a study protocol for developing a scalable model of interventions for moving towards the interruption of the transmission of soil-transmitted helminths and schistosome infections in the Wolaita zone of Ethiopia. Parasit Vectors. 2019;12(1):503. Epub 2019/10/31. doi: 10.1186/s13071-019-3757-4 ; PubMed Central PMCID: PMC6820996. 31665080 PMC6820996

[101] Means AR, Ajjampur SSR, Bailey R, Galactionova K, Gwayi-Chore MC, Halliday K, et al. Evaluating the sustainability, scalability, and replicability of an STH transmission interruption intervention: The DeWorm3 implementation science protocol. PLoS Negl Trop Dis. 2018;12(1):e0005988. Epub 2018/01/19. doi: 10.1371/journal.pntd.0005988 ; PubMed Central PMCID: PMC5773078. 29346376 PMC5773078

[102] Ásbjörnsdóttir KH, Ajjampur SSR, Anderson RM, Bailey R, Gardiner I, Halliday KE, et al. Assessing the feasibility of interrupting the transmission of soil-transmitted helminths through mass drug administration: The DeWorm3 cluster randomized trial protocol. PLoS Negl Trop Dis. 2018;12(1):e0006166. Epub 2018/01/19. doi: 10.1371/journal.pntd.0006166 ; PubMed Central PMCID: PMC5773085 29346377 PMC5773085

[103] World Health Organization. 2030 targets for soil-transmitted helminthiases control programmes. Geneva. 2019. Licence: CC BY-NC-SA 3.0 IGO.

