# Supplementary material for: Serological diagnosis of soil-transmitted helminth (Ascaris, Trichuris and hookworm) infections: A scoping review
Source: PLoS Negl Trop Dis. 2024 Apr 4;18(4):e0012049. doi: 10.1371/journal.pntd.0012049 (PMC10994556; doi:10.1371/journal.pntd.0012049)
Supplement: S2 Info — Our search strategy comprised two distinct components: (i) a literature search for published records and (ii) an exploration of commercially available assays and diagnostic services, and patents that either resulted in or could potentially result in commercial assays. (PDF) [file pntd.0012049.s002.pdf]

Serological diagnosis of soil-transmitted helminth (*Ascaris*, *Trichuris* and hookworm) infections: a scoping review

Sara Roose[1]*, Fiona Vande Velde[1], Johnny Vlaminck[1], Peter Geldhof[1], Bruno Levecke[1]*

[1] Department of Translational Physiology, Infectiology and Public Health, Ghent University, Merelbeke, Belgium
* Corresponding authors: Bruno.levecke@ugent.be (BL), sara.roose@ugent.be (SR)

Supporting information 2 - Detailed search strategy

1. Literature search

1.1 Ovid MEDLINE

Ovid MEDLINE(R) and Epub Ahead of Print, In-Process, In-Data-Review & Other Non-Indexed Citations and Daily <1946 to September 23, 2022>

1	(Serol* or seroprev* or seropos* or seroneg* or serodiagn*).tw.
2	(ELISA or enzyme?linked?immunosorbent?assay* or lateral?flow?assay* or LFA or point?of?care or POC or multiplex or western?blot or rapid?diagnostic?test or RDT or immunoassay* or diagnos*).tw.
3	(Blood or serum or plasma).tw.
4	(STH or soil?transmitted?helminth* or geohelminth*).tw.
5	(Ascari* or roundworm*).tw.
6	(Trichuri* or whipworm*).tw.
7	(Hookworm* or An#ylostoma or Necator).tw.
8	2 and 3
9	1 or 8
10	4 or 5 or 6 or 7
11	9 and 10

Ovid MEDLINE(R) and Epub Ahead of Print, In-Process, In-Data-Review & Other Non-Indexed Citations and Daily <1946 to October 11, 2022>

1	(Ascar* or Trichur* or hookworm*).mp.
2	((antigen* or immunogen*) adj2 (protein* or peptid*)).mp.
3	1 and 2

1.2 Embase

Sources Embase, MEDLINE, Preprints
1946 to September 23, 2022

Query (serol*:ab,ti OR seroprev*:ab,ti OR seropos*:ab,ti OR seroneg*:ab,ti OR serodiagn*:ab,ti OR ((elisa:ab,ti OR enzyme*linked*immunosorbent*assay*:ab,ti OR lateral*flow*assay*:ab,ti OR lfa:ab,ti OR point*of*care:ab,ti OR poc:ab,ti OR multiplex:ab,ti OR western*blot:ab,ti OR rapid*diagnostic*test:ab,ti OR rdt:ab,ti OR immunoassay*:ab,ti OR diagnos*:ab,ti) AND (blood:ab,ti OR serum:ab,ti OR plasma:ab,ti))) AND (sth:ab,ti OR soil?transmitted?helminth*:ab,ti OR geohelminth*:ab,ti OR ascari*:ab,ti OR roundworm*:ab,ti OR trichuri*:ab,ti OR whipworm*:ab,ti OR hookworm*:ab,ti OR an?ylostoma:ab,ti OR necator:ab,ti)

Mapped terms n/a

1.3 Cochrane Library

Date Run: 24/09/2022 19:24:19

#1 (Serol* OR seroprev* OR seropos* OR seroneg* OR serodiagn*):ab,ti
#2 (ELISA or enzyme*linked*immunosorbent*assay* or lateral*flow*assay* or LFA or point*of*care or POC or multiplex or western*blot or rapid*diagnostic*test or RDT or immunoassay* or diagnos*):ab,ti
#3 (Blood or serum or plasma):ab,ti
#4 (STH or soil?transmitted?helminth* or geohelminth*):ab,ti
#5 (Ascari* or roundworm*):ab,ti
#6 (Trichuri* or whipworm*):ab,ti
#7 (Hookworm* or An?ylostoma or Necator):ab,ti
#8 #2 AND #3
#9 #1 OR #8
#10 #4 OR #5 OR #6 OR #7
#11 #9 AND #10

2. Patent search (Https://worldwide.espacenet.com)

Date search: 12/10/2022

(ta all "ascaris" OR ta all "ascariasis" OR ta all "Trichuris" OR ta all "trichuriasis" OR ta all "hookworm" OR ta all "necator" OR ta all "ancylostoma" OR ta all "ankylostoma") AND (ta any "blood" OR ta any "serum" OR ta any "plasma")

(ta all "ascaris" OR ta all "ascariasis" OR ta all "Trichuris" OR ta all "trichuriasis" OR ta all "hookworm" OR ta all "necator" OR ta all "ancylostoma" OR ta all "ankylostoma") AND (ta any "diagnostic" OR ta any "diagnose" OR ta any "diagnosis")

3. Google keyword search (https://www.google.com)

Ascaris
Ascariasis serology kit

Trichuris
Trichuriasis serology kit, *Trichuris* serology kit, *Trichuris* antibody serology kit, *Trichuris* antibody detection, *Trichuris* diagnostic kit, Whipworm serology

Hookworm
Hookworm serology kit, *Ancylostoma* serology, *Necator* serology
