# Supplementary material for: Serological diagnosis of soil-transmitted helminth (Ascaris, Trichuris and hookworm) infections: A scoping review
Source: PLoS Negl Trop Dis. 2024 Apr 4;18(4):e0012049. doi: 10.1371/journal.pntd.0012049 (PMC10994556; doi:10.1371/journal.pntd.0012049)
Supplement: S4 Info — The number of records by technology principle (page 1), target analyte detected (page 2) and analyte detection agent used (page 3). Records are presented in historical order by year of publication. For target analyte and analyte detection agent, we have clustered the results on an STH species level. In cases where a record used multiple options, the records was included in the count of all relevant options. FAT: fluorescent antibody test, ELISA: enzyme-linked immunosorbent assay, MFI: multiplex flow immunoassay, qSAT: quantitative suspension array technology, SDS-PAGE: sodium dodecyl sulfate polyacrylamide gel electrophoresis, REIA: reverse enzyme immunoassay, RIA: radioimmunoassay, Ab: antibody, Ig: immunoglobulin, IC: immunecomplex, ES: excretory/secretory, HW: hookworm, STH: soil-transmitted helminth. (PDF) [file pntd.0012049.s004.pdf]

S4 Info. Characteristics of the assays used for serodiagnosis of STHs considering the evolving landscape of research over time

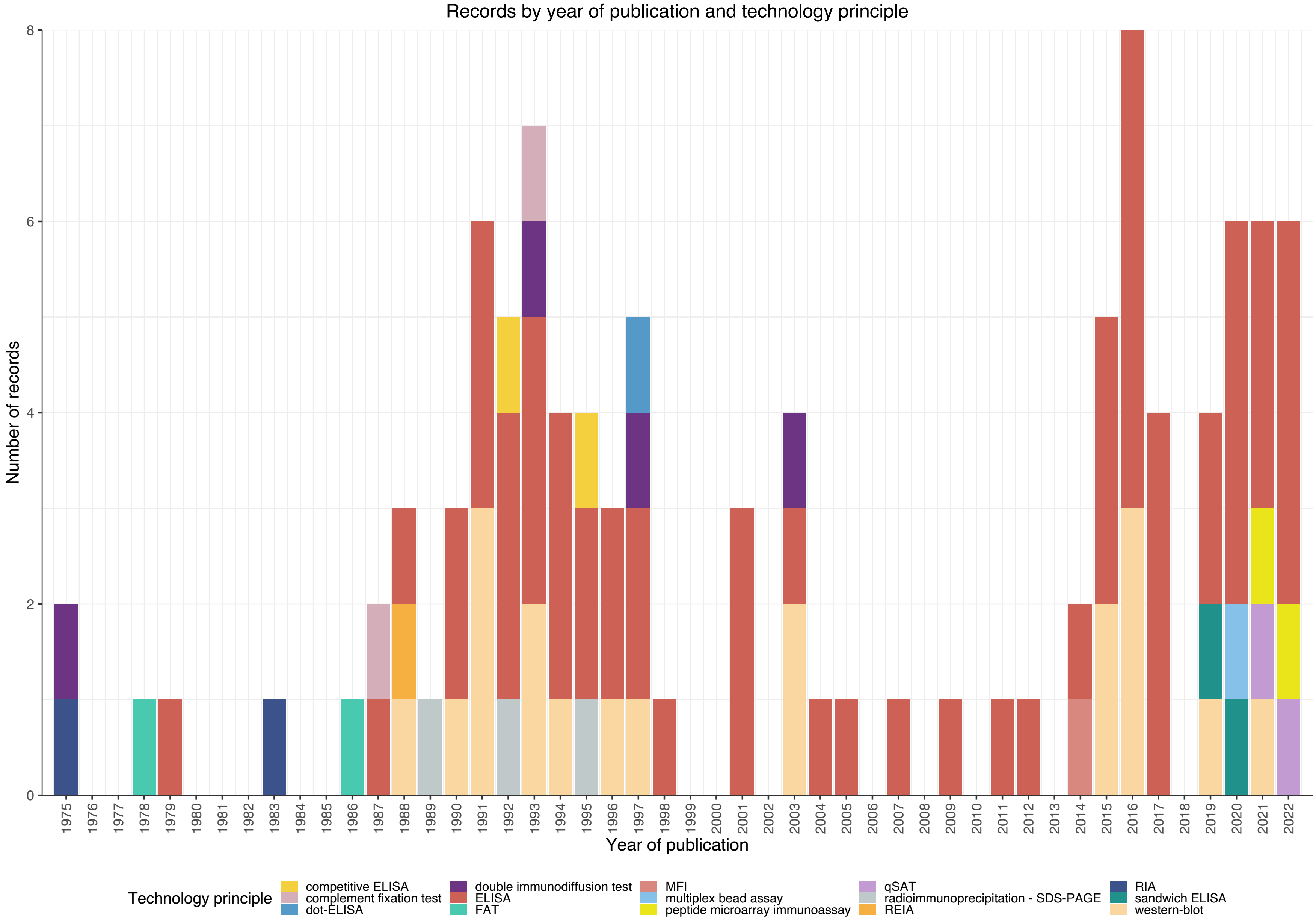

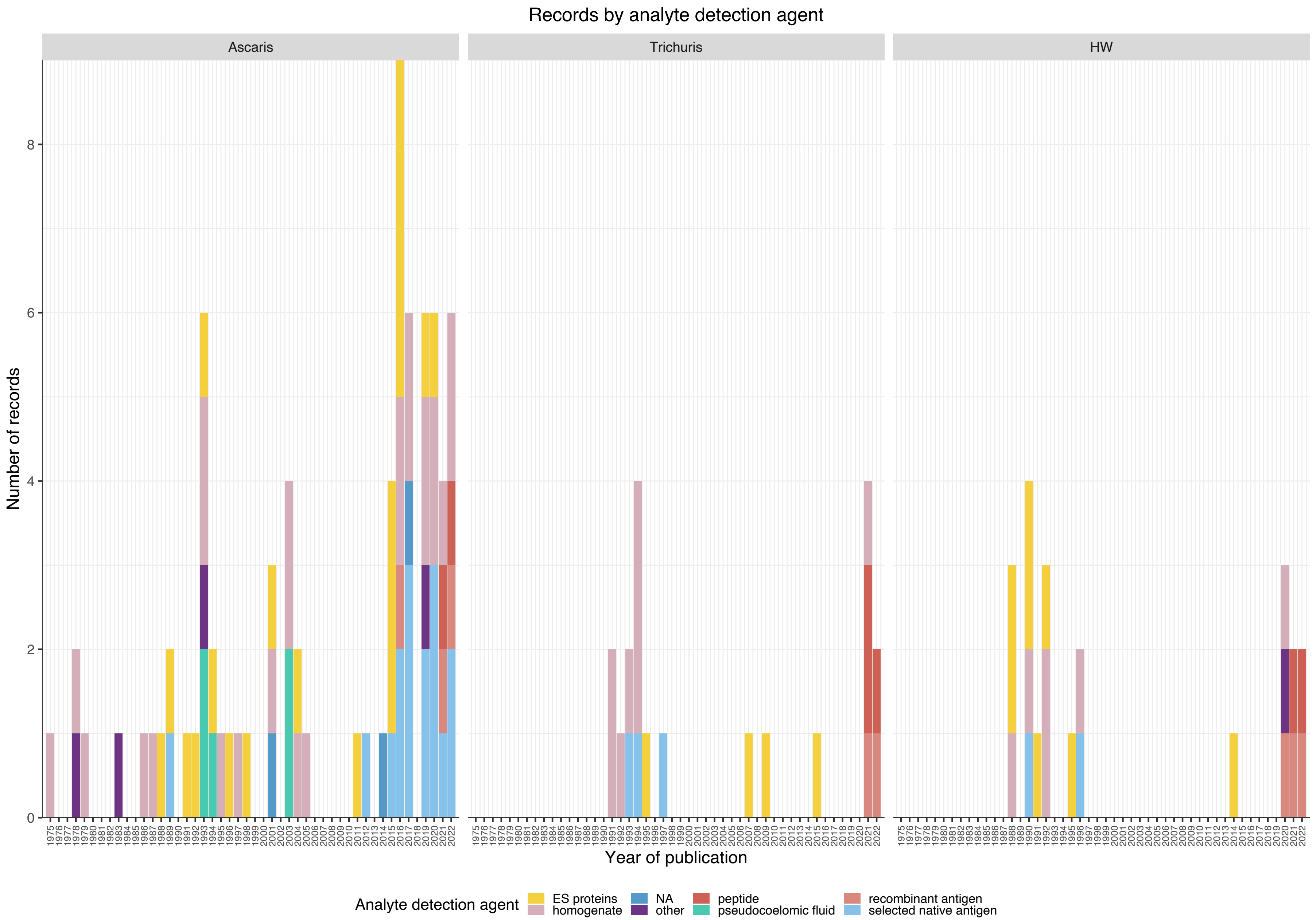
Records by analyte detection agent
Ascaris
Trichuris
HW
Number of records
Year of publication
Analyte detection agent
ES proteins
homogenate
NA
other
peptide
pseudocoelomic fluid
recombinant antigen
selected native antigen

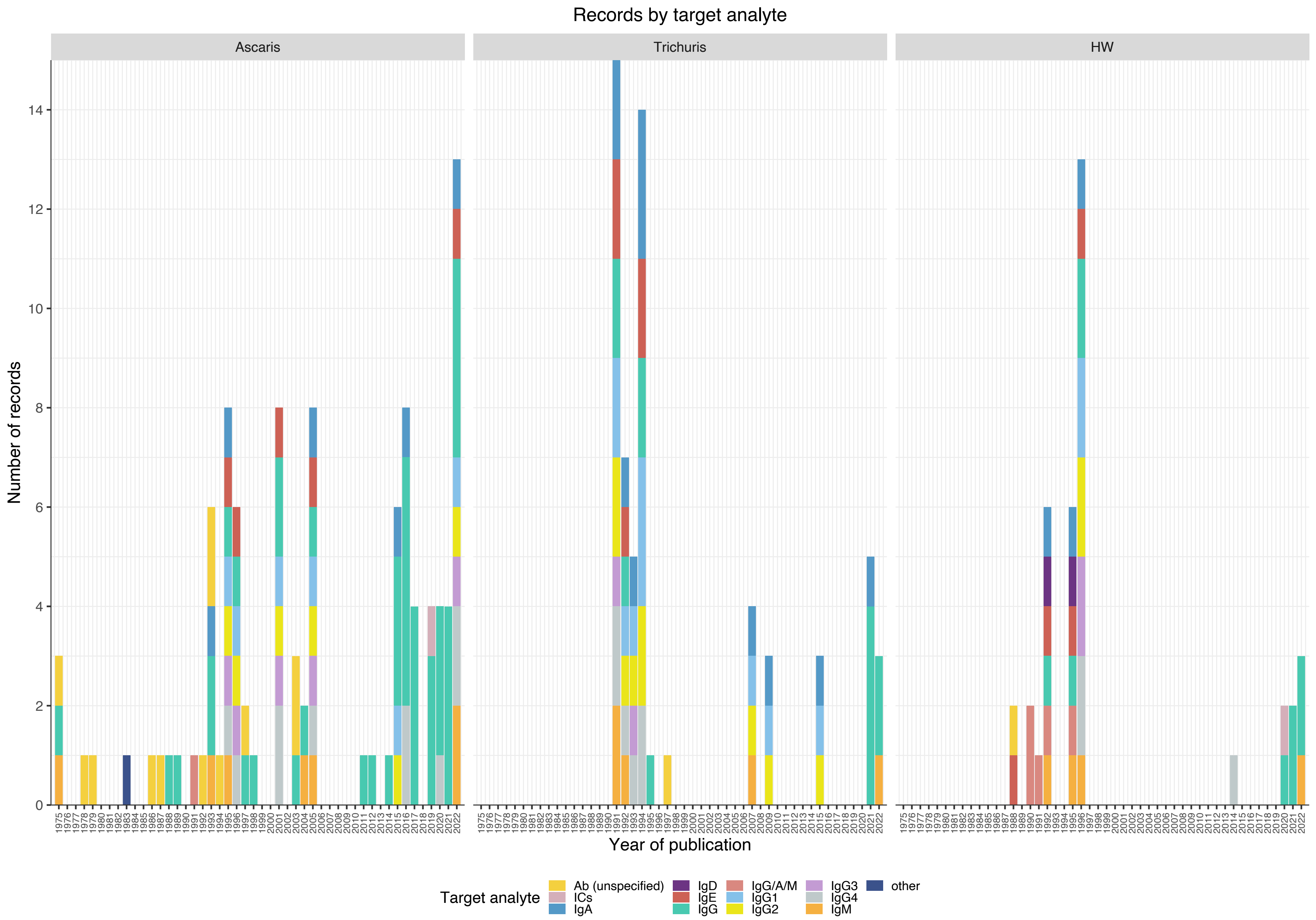
Records by target analyte
Ascaris
Trichuris
HW
Number of records
Year of publication
Target analyte
Ab (unspecified)
ICs
IgA
IgD
IgE
IgG
IgG/A/M
IgG1
IgG2
IgG3
IgG4
IgM
other
