# Supplementary material for: Serological diagnosis of soil-transmitted helminth (Ascaris, Trichuris and hookworm) infections: A scoping review
Source: PLoS Negl Trop Dis. 2024 Apr 4;18(4):e0012049. doi: 10.1371/journal.pntd.0012049 (PMC10994556; doi:10.1371/journal.pntd.0012049)
Supplement: S5 Info — References are presented in a table consistent with Fig 5 itself. (PDF) [file pntd.0012049.s005.pdf]

Serological diagnosis of soil-transmitted helminth (*Ascaris*, *Trichuris* and hookworm) infections: a scoping review

Sara Roose[1]*, Fiona Vande Velde[1], Johnny Vlaminck[1], Peter Geldhof[1], Bruno Levecke[1]*

[1] Department of Translational Physiology, Infectiology and Public Health, Ghent University, Merelbeke, Belgium
* Corresponding authors: Bruno.levecke@ugent.be (BL), sara.roose@ugent.be (SR)

Supporting information 5 - Overview of the records used to create Fig 5

Ascaris

	Stage 1	Stage 2	Stage 3	Stage 4
**Homogenate**	[1-15]	[1, 8, 10-19]	[1, 11, 15-25] + NovaLisa™ *Ascaris lumbricoides* IgG ELISA (NovaTec Immundiagnostica)	-
**ES proteins**	[9, 10, 14, 26-35]	[10, 14, 26, 30, 32-34, 36]	[30, 34-42]	-
**Pseudocoelomic fluid**	[4, 7, 12, 26, 27]	[12, 26]	-	-
**Selected native antigen**	[11, 31, 33, 43-45]	[11, 18, 33, 43-45]	[11, 18, 21-23, 43, 45-51] + SERASCA®-Test (Ghent University)	MonoScreen AbELISA *Ascaris suum* (Bio-X Diagnostics S.A.) + SERASCA®-Test (Ghent University)
**Recombinant antigen**	[45, 52]	[45, 52, 53]	[45, 53]	-
**Peptide**	[54, 55]	[54, 55]	-	-
**Other**	[5, 27, 56, 57]	[56]	-	-

Trichuris

	Stage 1	Stage 2	Stage 3	Stage 4
**Homogenate**	[58-61]	-	[61-65]	-
**ES proteins**	[66, 67]	-	[42, 68]	-
**Selected native antigen**	[60, 69]	[69]	[62]	-
**Recombinant antigen**	[52]	[52, 53]	[53]	-
**Peptide**	[54, 55, 58, 70]	[54, 55, 58]	-	-

Hookworm

	Stage 1	Stage 2	Stage 3	Stage 4
**Homogenate**	[71-75] [76]	[71, 72]	[73, 74]	-
**ES proteins**	[35, 71, 73, 76-79]	[71, 77]	[35, 73, 80]	-
**Selected native antigen**	[80, 81]	-	[80]	-
**Recombinant antigen**	[52, 72]	[52, 53, 72]	[53]	-
**Peptide**	[54, 55]	[54, 55]	-	-
**Other**	[82]	[82]	-	-

Three records were excluded from **Fig 5** ([83], [84] and [85]) because they made use of commercially available tests for which the analyte detection agent was unspecified.

References

1. Welch JS, Dobson C, Chopra S. Immunodiagnosis of Entamoeba histolytica and Ascaris lumbricoides infections in Caucasian and Aboriginal Australians. Transactions of the Royal Society of Tropical Medicine and Hygiene. 1986;80(2):240-7. doi: https://doi.org/10.1016/0035-9203(86)90024-6.
2. Speiser F, Weiss N. Comparative evaluation of 7 helminth antigens in the enzyme-linked immunosorbent assay (E.L.I.S.A.). Experientia. 1979;35(11):1512-4.
3. Yoshihara S, Nakagawa M, Suda H. Detection of complement fixation antibody against Ascaris suum antigen in pigs with white spots in the liver. Nihon Juigaku Zasshi. 1987;49(3):559-61. Epub 1987/06/01. doi: 10.1292/jvms1939.49.559. PubMed PMID: 3613356.
4. Takashima M, Ohmi H, Watanabe T, Okamoto K, Kanoe M, Nagai S. Attempts to separate female Ascaris suum antigen and to investigate its partial characterization. Veterinary journal (London, England : 1997). 2003;165(2):164-8.
5. Welch JS, Dobson C. Immunodiagnosis of parasitic zoonoses: comparative efficacy of three immunofluorescence tests using antigens purified by affinity chromatography. Transactions of the Royal Society of Tropical Medicine and Hygiene. 1978;72(3):282-8.
6. Maruyama H, Noda S, Choi W-Y, Ohta N, Nawa Y. Fine binding specificities to Ascaris suum and Ascaris lumbricoides antigens of the sera from patients of probable visceral larva migrans due to Ascaris suum. Parasitology International. 1997;46(3):181-8. doi: https://doi.org/10.1016/S1383-5769(97)00025-1.
7. Frontera E, Serrano F, Reina D, Alcaide M, Sanchez-Lopez J, Navarrete I. Serological responses to Ascaris suum adult worm antigens in Iberian finisher pigs. Journal of helminthology. 2003;77(2):167-72.
8. Patterson R, Huntley CC, Roberts M, Irons JS. Visceral larva migrans: Immunoglobulins, precipitating antibodies and detection of IgG and IgM antibodies against Ascaris antigen. The American journal of tropical medicine and hygiene. 1975;24(3):465-70.
9. Nguyen YTH, Hayata Y, Sonoda S, Nonaka N, Maruyama H, Yoshida A. Establishment of a serodiagnosis system for the detection of Toxocara spp. and Ascaris suum infection in chickens. Parasitology international. 2020;75:102022. doi: https://dx.doi.org/10.1016/j.parint.2019.102022.
10. Yoshida A, Kikuchi T, Nakagaki S, Maruyama H. Optimal ELISA antigen for the diagnosis of Ascaris suum infection in humans. Parasitology research. 2016;115(12):4701-5.
11. Vandekerckhove E, Vlaminck J, Geldhof P. Evaluation of serology to measure exposure of piglets to Ascaris suum during the nursery phase. Veterinary parasitology. 2017;246:82-7. doi: https://dx.doi.org/10.1016/j.vetpar.2017.09.008.
12. Yoshihara S, Oya T, Furuya T, Goto N. Use of body fluid of adult female Ascaris suum as an antigen in the enzyme-linked immunosorbent assay (ELISA) for diagnosis of swine ascariosis. Journal of helminthology. 1993;67(4):279-86.
13. Bhattacharyya T, Santra A, Majumder DN, Chatterjee BP. Possible approach for serodiagnosis of ascariasis by evaluation of immunoglobulin G4 response using Ascaris lumbricoides somatic antigen. Journal of clinical microbiology. 2001;39(8):2991-4.
14. Fan C-K, Su K-E. Cross-reactions with Ascaris suum antigens of sera from mice infected with A. suum, Toxocara canis, and Angiostrongylus cantonensis. Parasitology international. 2004;53(3):263-71.
15. Dana D, Roose S, Vlaminck J, Ayana M, Mekonnen Z, Geldhof P, et al. Longitudinal assessment of the exposure to Ascaris lumbricoides through copromicroscopy and serology in school children from Jimma Town, Ethiopia. PLoS neglected tropical diseases. 2022;16(1):e0010131. doi: https://dx.doi.org/10.1371/journal.pntd.0010131.
16. Lynch NR, Hagel I, Vargas V, Rotundo A, Varela MC, Di Prisco MC, et al. Comparable seropositivity for ascariasis and toxocariasis in tropical slum children. Parasitology research. 1993;79(7):547-50.
17. de-la-Rosa-Arana J-L, Ponce-Noguez J-B, Reyes-Rodriguez N-E, Vega-Sanchez V, Zepeda-Velazquez A-P, Martinez-Juarez V-M, et al. Helminths of the Wild Boar (Sus scrofa) from Units of Conservation Management and Sustainable Use of Wildlife Installed in the Eastern Economic Region of Mexico. Animals : an open access journal from MDPI. 2021;11(1). doi: https://dx.doi.org/10.3390/ani11010098.
18. Dana D, Vlaminck J, Ayana M, Tadege B, Mekonnen Z, Geldhof P, et al. Evaluation of copromicroscopy and serology to measure the exposure to Ascaris infections across age groups and to assess the impact of 3 years

of biannual mass drug administration in Jimma Town, Ethiopia. PLoS neglected tropical diseases. 2020;14(4):e0008037. doi: https://dx.doi.org/10.1371/journal.pntd.0008037.
19. Roose S, Leta GT, Vlaminck J, Getachew B, Mekete K, Peelaers I, et al. Comparison of coproprevalence and seroprevalence to guide decision-making in national soil-transmitted helminthiasis control programs: Ethiopia as a case study. PLoS Negl Trop Dis. 2022;16(10):e0010824. Epub 2022/10/06. doi: 10.1371/journal.pntd.0010824. PubMed PMID: 36197895; PubMed Central PMCID: PMCPMC9534397.
20. Lassen B, Janson M, Viltrop A, Neare K, Hutt P, Golovljova I, et al. Serological Evidence of Exposure to Globally Relevant Zoonotic Parasites in the Estonian Population. PloS one. 2016;11(10):e0164142. doi: https://dx.doi.org/10.1371/journal.pone.0164142.
21. Martinez-Perez JM, Vandekerckhove E, Vlaminck J, Geldhof P, Martinez-Valladares M. Serological detection of Ascaris suum at fattening pig farms is linked with performance and management indices. Veterinary parasitology. 2017;248:33-8. doi: https://dx.doi.org/10.1016/j.vetpar.2017.10.009.
22. Vandekerckhove E, Vlaminck J, Sacristan RDP, Geldhof P. Effect of strategic deworming on Ascaris suum exposure and technical performance parameters in fattening pigs. Veterinary parasitology. 2019;268:67-72. doi: https://dx.doi.org/10.1016/j.vetpar.2019.03.006.
23. Lassen B, Geldhof P, Halli O, Vlaminck J, Oliviero C, Orro T, et al. Anti-Ascaris suum IgG antibodies in fattening pigs with different respiratory conditions. Veterinary parasitology. 2019;265:85-90. doi: https://dx.doi.org/10.1016/j.vetpar.2018.12.005.
24. Palmer DR, Hall A, Haque R, Anwar KS. Antibody isotype responses to antigens of Ascaris lumbricoides in a case-control study of persistently heavily infected Bangladeshi children. Parasitology. 1995;111 (Pt 3):385-93.
25. King EM, Kim HT, Dang NT, Michael E, Drake L, Needham C, et al. Immuno-epidemiology of Ascaris lumbricoides infection in a high transmission community: antibody responses and their impact on current and future infection intensity. Parasite Immunol. 2005;27(3):89-96. Epub 2005/05/11. doi: 10.1111/j.1365-3024.2005.00753.x. PubMed PMID: 15882235.
26. Bøgh HO, Eriksen L, Lawson LG, Lind P. Evaluation of an enzyme-linked immunosorbent assay and a histamine release test system for the detection of pigs naturally infected with Ascaris suum. Preventive Veterinary Medicine. 1994;21(3):201-14. doi: https://doi.org/10.1016/0167-5877(94)90019-1.
27. Lind P, Eriksen L, Nansen P, Nilsson O, Roepstorff A. Response to repeated inoculations with Ascaris suum eggs in pigs during the fattening period. II. Specificf IgA, IgG, and IgM antibodies determined by enzyme-linked immunosorbent assay. Parasitology Research. 1993;79(3):240-4.
28. Schneider R, Obwaller A, Auer H. Immunoblot for the detection of Ascaris suum-specific antibodies in patients with visceral larva migrans (VLM) syndrome. Parasitology research. 2015;114(1):305-10. doi: https://dx.doi.org/10.1007/s00436-014-4196-y.
29. Boyce WM, Branstetter BA, Kazacos KR. Comparative analysis of larval excretory-secretory antigens of Baylisascaris procyonis, Toxocara canis and Ascaris suum by Western blotting and enzyme immunoassay. Int J Parasitol. 1988;18(1):109-13. Epub 1988/02/01. doi: 10.1016/0020-7519(88)90044-6. PubMed PMID: 3366527.
30. Roepstorff A. Natural Ascaris suum infections in swine diagnosed by coprological and serological (ELISA) methods. Parasitology research. 1998;84(7):537-43.
31. Haswell-Elkins MR, Kennedy MW, Maizels RM, Elkins DB, Anderson RM. The antibody recognition profiles of humans naturally infected with Ascaris lumbricoides. Parasite Immunol. 1989;11(6):615-27. Epub 1989/11/01. doi: 10.1111/j.1365-3024.1989.tb00925.x. PubMed PMID: 2616191.
32. Chatterjee BP, Santra A, Karmakar PR, Mazumder DN. Evaluation of IgG4 response in ascariasis by ELISA for serodiagnosis. Tropical medicine & international health : TM & IH. 1996;1(5):633-9.
33. Vlaminck J, Masure D, Wang T, Nejsum P, Hokke CH, Geldhof P. A Phosphorylcholine-Containing Glycolipid-like Antigen Present on the Surface of Infective Stage Larvae of Ascaris spp. Is a Major Antibody Target in Infected Pigs and Humans. PLoS neglected tropical diseases. 2016;10(12):e0005166. doi: https://dx.doi.org/10.1371/journal.pntd.0005166.
34. Santra A, Bhattacharya T, Chowdhury A, Ghosh A, Ghosh N, Chatterjee BP, et al. Serodiagnosis of ascariasis with specific IgG4 antibody and its use in an epidemiological study. Transactions of the Royal Society of Tropical Medicine and Hygiene. 2001;95(3):289-92.
35. Pritchard DI, Quinnell RJ, McKean PG, Walsh L, Leggett KV, Slater AF, et al. Antigenic cross-reactivity between Necator americanus and Ascaris lumbricoides in a community in Papua New Guinea infected predominantly with hookworm. Trans R Soc Trop Med Hyg. 1991;85(4):511-4. Epub 1991/07/01. doi: 10.1016/0035-9203(91)90239-u. PubMed PMID: 1755061.
36. Pinelli E, Herremans T, Harms MG, Hoek D, Kortbeek LM. Toxocara and Ascaris seropositivity among patients suspected of visceral and ocular larva migrans in the Netherlands: trends from 1998 to 2009. European

journal of clinical microbiology & infectious diseases : official publication of the European Society of Clinical Microbiology. 2011;30(7):873-9. doi: https://dx.doi.org/10.1007/s10096-011-1170-9.
37. Haswell-Elkins MR, Leonard H, Kennedy MW, Elkins DB, Maizels RM. Immunoepidemiology of Ascaris lumbricoides: relationships between antibody specificities, exposure and infection in a human community. Parasitology. 1992;104 Pt 1:153-9.
38. Araujo Z, Brandes S, Pinelli E, Bochichio MA, Palacios A, Wide A, et al. Seropositivity for ascariosis and toxocariosis and cytokine expression among the indigenous people in the Venezuelan Delta region. Revista do Instituto de Medicina Tropical de Sao Paulo. 2015;57(1):47-55. doi: https://dx.doi.org/10.1590/S0036-46652015000100007.
39. Schneider R, Auer H. Incidence of Ascaris suum-specific antibodies in Austrian patients with suspected larva migrans visceralis (VLM) syndrome. Parasitology research. 2016;115(3):1213-9. doi: https://dx.doi.org/10.1007/s00436-015-4857-5.
40. Mughini-Gras L, Harms M, van Pelt W, Pinelli E, Kortbeek T. Seroepidemiology of human Toxocara and Ascaris infections in the Netherlands. Parasitology research. 2016;115(10):3779-94. doi: https://dx.doi.org/10.1007/s00436-016-5139-6.
41. Taus K, Schmoll F, El-Khatib Z, Auer H, Holzmann H, Aberle S, et al. Occupational swine exposure and Hepatitis E virus, Leptospira, Ascaris suum seropositivity and MRSA colonization in Austrian veterinarians, 2017-2018-A cross-sectional study. Zoonoses and public health. 2019;66(7):842-51. doi: https://dx.doi.org/10.1111/zph.12633.
42. Kringel H, Thamsborg SM, Petersen HH, Göring HHH, Skallerup P, Nejsum P. Serum antibody responses in pigs trickle-infected with Ascaris and Trichuris: Heritabilities and associations with parasitological findings. Veterinary Parasitology. 2015;211(3):306-11. doi: https://doi.org/10.1016/j.vetpar.2015.06.008.
43. Njenga SM, Kanyi HM, Arnold BF, Matendechero SH, Onsongo JK, Won KY, et al. Integrated Cross-Sectional Multiplex Serosurveillance of IgG Antibody Responses to Parasitic Diseases and Vaccines in Coastal Kenya. The American journal of tropical medicine and hygiene. 2020;102(1):164-76. doi: https://dx.doi.org/10.4269/ajtmh.19-0365.
44. Vlaminck J, Nejsum P, Vangroenweghe F, Thamsborg SM, Vercruysse J, Geldhof P. Evaluation of a serodiagnostic test using Ascaris suum haemoglobin for the detection of roundworm infections in pig populations. Veterinary parasitology. 2012;189(2-4):267-73. doi: https://dx.doi.org/10.1016/j.vetpar.2012.04.024.
45. Vlaminck J, Supali T, Geldhof P, Hokke CH, Fischer PU, Weil GJ. Community Rates of IgG4 Antibodies to Ascaris Haemoglobin Reflect Changes in Community Egg Loads Following Mass Drug Administration. PLoS neglected tropical diseases. 2016;10(3):e0004532. doi: https://dx.doi.org/10.1371/journal.pntd.0004532.
46. Lassen B, Oliviero C, Orro T, Jukola E, Laurila T, Haimi-Hakala M, et al. Effect of fenbendazole in water on pigs infected with Ascaris suum in finishing pigs under field conditions. Veterinary parasitology. 2017;237:1-7. doi: https://dx.doi.org/10.1016/j.vetpar.2017.03.005.
47. Joachim A, Winkler C, Ruczizka U, Ladinig A, Koch M, Tichy A, et al. Comparison of different detection methods for Ascaris suum infection on Austrian swine farms. Porcine health management. 2021;7(1):57. doi: https://dx.doi.org/10.1186/s40813-021-00236-9.
48. Delsart M, Fablet C, Rose N, Reperant J-M, Blaga R, Dufour B, et al. Descriptive epidemiology of the main internal parasites on alternative pig farms in france. The Journal of parasitology. 2022;108(4):306-21. doi: https://dx.doi.org/10.1645/21-126.
49. Tassis P, Symeonidou I, Gelasakis AI, Kargaridis M, Aretis G, Arsenopoulos KV, et al. Serological Assessment of Ascaris suum Exposure in Greek Pig Farms and Associated Risk Factors Including Lawsonia intracellularis. Pathogens (Basel, Switzerland). 2022;11(9). doi: https://dx.doi.org/10.3390/pathogens11090959.
50. Vlaminck J, Dusseldorf S, Heres L, Geldhof P. Serological examination of fattening pigs reveals associations between Ascaris suum, lung pathogens and technical performance parameters. Veterinary parasitology. 2015;210(3-4):151-8. doi: https://dx.doi.org/10.1016/j.vetpar.2015.04.012.
51. Zheng Y, Xie Y, Geldhof P, Vlaminck J, Ma G, Gasser RB, et al. High anti-Ascaris seroprevalence in fattening pigs in Sichuan, China, calls for improved management strategies. Parasites & vectors. 2020;13(1):60. doi: https://dx.doi.org/10.1186/s13071-020-3935-4.
52. Santano R, Rubio R, Grau-Pujol B, Escola V, Muchisse O, Cuamba I, et al. Evaluation of antibody serology to determine current helminth and Plasmodium falciparum infections in a co-endemic area in Southern Mozambique. PLoS neglected tropical diseases. 2022;16(6):e0010138. doi: https://dx.doi.org/10.1371/journal.pntd.0010138.

53. Santano R, Rubio R, Grau-Pujol B, Escola V, Muchisse O, Cuamba I, et al. Plasmodium falciparum and Helminth Coinfections Increase IgE and Parasite-Specific IgG Responses. Microbiology spectrum. 2021;9(3):e0110921. doi: https://dx.doi.org/10.1128/Spectrum.01109-21.
54. Vlaminck J, Lagatie O, Dana D, Mekonnen Z, Geldhof P, Levecke B, et al. Identification of antigenic linear peptides in the soil-transmitted helminth and Schistosoma mansoni proteome. PLoS Negl Trop Dis. 2021;15(4):e0009369. Epub 2021/04/29. doi: 10.1371/journal.pntd.0009369. PubMed PMID: 33909616.
55. Vengesai A, Naicker T, Midzi H, Kasambala M, Mduluza-Jokonya TL, Rusakaniko S, et al. Multiplex peptide microarray profiling of antibody reactivity against neglected tropical diseases derived B-cell epitopes for serodiagnosis in Zimbabwe. PloS one. 2022;17(7):e0271916. doi: https://dx.doi.org/10.1371/journal.pone.0271916.
56. Lopes CA, de Faria LS, de Sousa JEN, Borges IP, Ribeiro RP, Bueno LL, et al. Anti-Ascaris suum immunoglobulin Y as a novel biotechnological tool for the diagnosis of human ascariasis. Journal of helminthology. 2019;94:e71. doi: https://dx.doi.org/10.1017/S0022149X19000701.
57. Tanaka K, Kawamura H, Tohgi N, Tsuji M, Miyachi Y, Miyoshi A. The measurement of Ascaris suum protein by radioimmunoassay in sera from patients with helminthiasis and with gastrointestinal diseases. Parasitology. 1983;86 (Pt 2):291-300.
58. Cruz K, Marcilla A, Kelly P, Vandenplas M, Osuna A, Trelis M. Trichuris trichiura egg extract proteome reveals potential diagnostic targets and immunomodulators. PLoS neglected tropical diseases. 2021;15(3):e0009221. doi: https://dx.doi.org/10.1371/journal.pntd.0009221.
59. Lillywhite JE, Bundy DA, Didier JM, Cooper ES, Bianco AE. Humoral immune responses in human infection with the whipworm Trichuris trichiura. Parasite Immunol. 1991;13(5):491-507. Epub 1991/09/01. doi: 10.1111/j.1365-3024.1991.tb00546.x. PubMed PMID: 1956697.
60. Needham CS, Lillywhite JE, Didier JM, Bianco AE, Bundy DAP. Age-dependency of serum isotype responses and antigen recognition in human whipworm (Trichuris trichiura) infection. Parasite Immunology. 1993;15(12):683-92. doi: https://doi.org/10.1111/j.1365-3024.1993.tb00583.x.
61. Needham CS, Lillywhite JE, Didier JM, Bianco AE, Bundy DAP. Temporal changes in Trichuris trichiura infection intensity and serum isotype responses in children. Parasitology. 1994;109(2):197-200. Epub 2009/04/06. doi: 10.1017/S0031182000076307.
62. Needham CS, Lillywhite JE, Didier JM, Bianco AT, Bundy DAP. Comparison of age-dependent antigen recognition in two communities with high and low Trichuris trichiura transmission. Acta Tropica. 1994;58(2):87-98. doi: https://doi.org/10.1016/0001-706X(94)90048-5.
63. Needham CS, Lillywhite JE, Didier JM, Bundy DAP. Serum isotype responses after treatment of human trichuriasis. Transactions of The Royal Society of Tropical Medicine and Hygiene. 1994;88(3):354-5. doi: 10.1016/0035-9203(94)90114-7.
64. Bundy DA, Lillywhite JE, Didier JM, Simmons I, Bianco AE. Age-dependency of infection status and serum antibody levels in human whipworm (Trichuris trichiura) infection. Parasite immunology. 1991;13(6):629-38.
65. Needham CS, Bundy DAP, Lillywhite JE, Didier JM, Simmons I, Bianco AE. The relationship between Trichuris trichiura transmission intensity and the age-profiles of parasite-specific antibody isotypes in two endemic communities. Parasitology. 1992;105(2):273-83. Epub 2009/04/06. doi: 10.1017/S0031182000074205.
66. Lillywhite JE, Cooper ES, Needham CS, Venugopal S, Bundy DAP, Bianco AE. Identification and characterization of excreted/secreted products of Trichuris trichiura. Parasite Immunology. 1995;17(1):47-54. doi: https://doi.org/10.1111/j.1365-3024.1995.tb00965.x.
67. Kringel H, Roepstorff A. Trichuris suis excretory/secretory antigen-specific antibodies in serum from single-inoculated pigs. Parasite immunology. 2007;29(6):327-30.
68. Nejsum P, Thamsborg SM, Petersen HH, Kringel H, Fredholm M, Roepstorff A. Population dynamics of Trichuris suis in trickle-infected pigs. Parasitology. 2009;136(6):691-7. Epub 2009/04/16. doi: 10.1017/S0031182009005976.
69. Hill DE, Romanowski RD, Urban JF, Jr. A Trichuris specific diagnostic antigen from culture fluids of Trichuris suis adult worms. Veterinary parasitology. 1997;68(1-2):91-102.
70. Cruz K. Trichuris trichiura: Assessment of potential alternative diagnostic methods and efficacy of treatments in the African Green Monkey animal model (Chlorocebus sabaeus) [dissertation]. 2021.
71. Shetty P, Dilawari JB, Virk KJ, Sehgal R, Ganguly NK, Mahajan RC. Enzyme-linked immunosorbent assay using excretory/secretory and somatic antigens as a diagnostic test for human hookworm infection. Transactions of the Royal Society of Tropical Medicine and Hygiene. 1988;82(5):736-8.
72. Logan J, Pearson MS, Manda SS, Choi Y-J, Field M, Eichenberger RM, et al. Comprehensive analysis of the secreted proteome of adult Necator americanus hookworms. PLoS neglected tropical diseases. 2020;14(5):e0008237. doi: https://dx.doi.org/10.1371/journal.pntd.0008237.

73. Pritchard DI, Walsh EA, Quinell RJ, Raiko A, Edmonds P, Keymer AE. Isotypic variation in antibody responses in a community in Papua New Guinea to larval and adult antigens during infection, and following reinfection, with the hookworm Necator americanus. Parasite Immunol. 1992;14(6):617-31. Epub 1992/11/01. doi: 10.1111/j.1365-3024.1992.tb00034.x. PubMed PMID: 1470481.
74. Palmer. IgG4 responses to antigens of adult Necator americanus: potential for use in large-scale epidemiological studies. 1996. Bulletin of the World Health Organization, 1996, 74 (4): 381-386
75. Timothy LM, Coulson PS, Behnke JM, Wilson RA. Cross-reactivity between Necator americanus and Schistosoma mansoni in mice. International journal for parasitology. 1992;22(8):1143-9.
76. Pritchard DI, Mckean PG, Rogan MT, Schad GA. The identification of a species-specific antigen from Necator americanus. Parasite Immunology. 1990;12(3):259-67. doi: https://doi.org/10.1111/j.1365-3024.1990.tb00953.x.
77. Ganguly NK, Mahajan RC, Sehgal R, Shetty P, Dilawari JB. Role of specific immunoglobulin E to excretory-secretory antigen in diagnosis and prognosis of hookworm infection. Journal of clinical microbiology. 1988;26(4):739-42.
78. Pritchard DI, Walsh EA. The specificity of the human IgE response to Necator americanus. Parasite Immunology. 1995;17(11):605-7. doi: https://doi.org/10.1111/j.1365-3024.1995.tb01005.x.
79. DuVall AS, Fairley JK, Sutherland L, Bustinduy AL, Mungai PL, Muchiri EM, et al. Development of a specimen-sparing multichannel bead assay to detect antiparasite IgG4 for the diagnosis of Schistosoma and Wuchereria infections on the coast of Kenya. Am J Trop Med Hyg. 2014;90(4):638-45. Epub 2014/02/12. doi: 10.4269/ajtmh.13-0292. PubMed PMID: 24515945; PubMed Central PMCID: PMCPMC3973507.
80. Pritchard DI, Quinnell RJ, Slater AF, McKean PG, Dale DD, Raiko A, et al. Epidemiology and immunology of Necator americanus infection in a community in Papua New Guinea: humoral responses to excretory-secretory and cuticular collagen antigens. Parasitology. 1990;100 Pt 2:317-26. Epub 1990/04/01. doi: 10.1017/s0031182000061333. PubMed PMID: 2345664.
81. Loukas A, Opdebeeck J, Croese J, Prociv P. Immunoglobulin G subclass antibodies against excretory/secretory antigens of Ancylostoma caninum in human enteric infections. The American journal of tropical medicine and hygiene. 1996;54(6):672-6.
82. Souza DCd, de Faria LS, Sousa JENd, Lopes CdA, Ribeiro VdS, da Silva VJ, et al. Use of polyclonal IgY antibodies to detect serum immune complexes in patients with active hookworm infection. Parasitology. 2020;147(6):715-20. doi: https://dx.doi.org/10.1017/S0031182020000220.
83. Malafiej E, Spiewak E. Serological investigation in children infected with Ascaris lumbricoides. Wiadomosci parazytologiczne. 2001;47(4):585-90.
84. Fortunato S, Castagna B, Monteleone MR, Pierro R, Cringoli G, Bruschi F. Parasite prevalence in a village in Burkina Faso: the contribution of new techniques. Journal of infection in developing countries. 2014;8(5):670-5. doi: https://dx.doi.org/10.3855/jidc.3660.
85. Guo F, Forde MS, Werre SR, Krecek RC, Zhu G. Seroprevalence of five parasitic pathogens in pregnant women in ten Caribbean countries. Parasitology research. 2017;116(1):347-58.
