# Supplementary material for: Serological diagnosis of soil-transmitted helminth (Ascaris, Trichuris and hookworm) infections: A scoping review
Source: PLoS Negl Trop Dis. 2024 Apr 4;18(4):e0012049. doi: 10.1371/journal.pntd.0012049 (PMC10994556; doi:10.1371/journal.pntd.0012049)
Supplement: S6 Info — Records were retrieved by an Espacenet patent search. NA: information not available. (PDF) [file pntd.0012049.s006.pdf]

Serological diagnosis of soil-transmitted helminth (*Ascaris*, *Trichuris* and hookworm) infections: a scoping review

Sara Roose[1]*, Fiona Vande Velde[1], Johnny Vlaminck[1], Peter Geldhof[1], Bruno Levecke[1]*

[1] Department of Translational Physiology, Infectiology and Public Health, Ghent University, Merelbeke, Belgium
* Corresponding authors: Bruno.levecke@ugent.be (BL), sara.roose@ugent.be (SR)

Supporting information 6 - Overview of the patent documents related to serological diagnosis for soil-transmitted helminthiases

Title	Invention	Target species	Technology principle	Target analyte	Analyte-detection agent	Test species	Publication	Year
Nucleic acids encoding *Ancylostoma* secreted protein	Nucleic acid molecule for the recombinant production of ASP protein for vaccination, selective treatment and diagnostics	Hookworm	Any of the known methods for detection of antigens, nucleic acids, and antibodies	ASP, hookworm nucleic acid, and anti-ASP antibodies	*Ancylostoma* Secreted Protein (ASP) of 37-40 kDa	Human, dog	US5753787	1998
Immunological method for the diagnosis of avian ascariasis	In vitro method of diagnosing avian ascaridiasis and diagnostic kit	*Ascaris*	ELISA	IgG	complete *Ascaris suum* extract	Poultry	WO2005071415 A1	2005
Method of specific *Ascaris lumbricoides* detection in clinical material in various stages of disease	Highly specific and highly sensitive method for the specific detection of *Ascaris lumbricoides* at various, including early, stages of the disease	*Ascaris*	PCR	fragment of the 18S gene of ribosomal RNA (rRNA) of *Ascaris lumbricoides*	NA	Human	RU2424525C1	2011

Records were retrieved by an Espacenet patent search. NA: information not available.
